# Synthesis of Degradable Polyolefins Bearing Disulfide Units via Metathesis Copolymerization

**DOI:** 10.3390/polym15143101

**Published:** 2023-07-20

**Authors:** Yu Xia, Fulin Zhou, Wenyan Hao, Shan Tang

**Affiliations:** 1 College of Chemistry and Chemical Engineering, Jiangxi Normal University, Nanchang 330022, China; 2 Frontiers Science Center for Transformative Molecules, School of Chemistry and Chemical Engineering, Shanghai Jiao Tong University, Shanghai 200240, China

**Keywords:** disulfides, polyolefin, copolymerization, polymer degradation

## Abstract

Disulfide bonds are dynamic covalent bonds, which are easy to cleave and reform upon chemical stimulus. Various methods including the oxidative coupling of thiols and polymerization of disulfide-containing monomers have been developed for the synthesis of poly(disulfide)s. However, installing small amounts of disulfide units in the main chain of polyolefins has received much less attention. Herein, we report a novel strategy for incorporating cleavable disulfide units into the backbone of polyolefins using commercially available diallyl disulfide (DADS) as a comonomer via metathesis copolymerization. The copolymerization of diallyl disulfide with cyclooctene occurred using the second-generation Grubbs catalyst under mild conditions, allowing for the synthesis of copolymers with adjustable disulfide content ranging from 0.7 to 8.5 mol%, and the molecular weight of the obtained copolymers ranged from 5.8 kg·mol^−1^ to 42.8 kg·mol^−1^. The resulting polyolefins with disulfide insertion retained excellent thermal processability and exhibited degradability. Treatment of the copolymer (8.5 mol% disulfide content) with tri-*n*-butylphosphine resulted in a significant reduction in molecular weight from 5.8 kg·mol^−1^ to 1.6 kg·mol^−1^. Successful copolymerization with diallyl disulfide provides a convenient and effective method for obtaining degradable polyolefins.

## 1. Introduction

Disulfide bonds are dynamic covalent bonds, which have found wide application in areas such as self-sorting [1,2], biomolecular engineering [3], and biomembrane analysis [4]. They frequently serve as pendants or cross-linkers in the side chain to give star polymers [5], micelles [6], branched polymers [7,8], and gels [9]. For example, interchain disulfide bonds are essential for protein folding and stabilization of tertiary structures [10,11]. Since the cleavage and reformation of disulfide bonds are convenient and efficient upon chemical stimulus [12,13,14,15], the incorporation of disulfide bonds into the main chain of a polymer will make it easy to degrade and recycle. Hence, the development of concise and highly effective pathways to access poly(disulfide)s have received great attention over the past decades. Various methods including oxidative coupling of thiols [16,17,18,19] and polymerization of disulfide-containing monomers [8,20,21,22,23,24,25] have been developed for the synthesis of poly(disulfide)s. Besides the synthesis of poly(disulfide)s with repeating disulfides, little success has been made in incorporating a low content of disulfides into the main chain of commodity polymers [19,26].

**Scheme 1:**
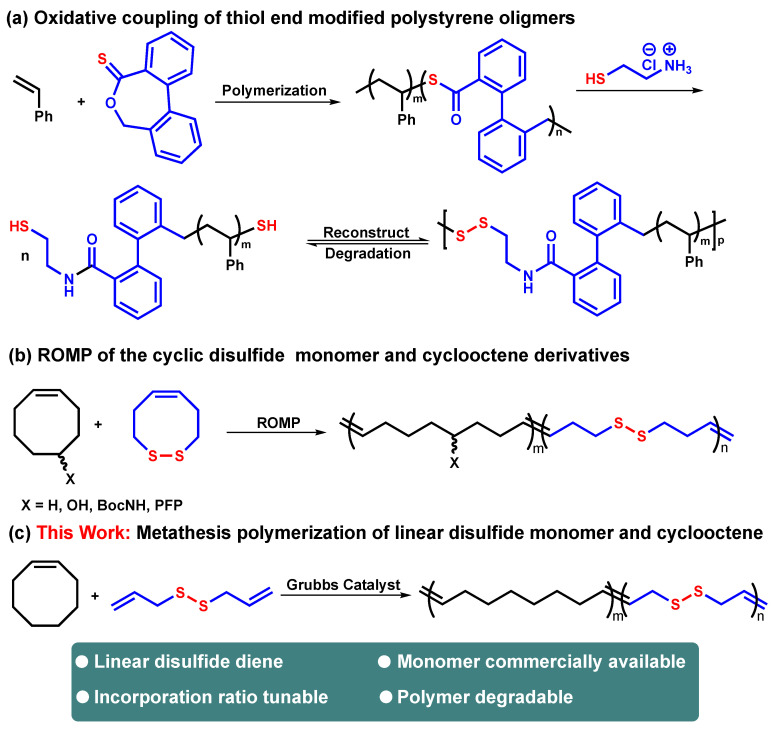
Methods for accessing polyolefins bearing low-content disulfide units. (**a**) Oxidative coupling of thiol end-modified polystyrene oligomers to access disulfide-containing polystyrenes; (**b**) Synthesis of disulfide-containing polyolefin by the ROMP of the cyclic disulfide monomer; (**c**) Synthesis of the disulfide-containing polyolefin by olefin metathesis polymerization of the linear acyclic disulfide monomer and cyclooctene.

Polyolefins are produced in the largest volume among all synthetic polymers [27,28], whereas the chemical inertness of the carbon–carbon chains prevents them from efficient chemical recycling [29]. In recent years, there has been a growing interest in the synthesis of polyolefin copolymers that possess both desirable material properties and degradability [30,31,32,33,34,35,36]. As disulfide bonds are dynamic covalent bonds, installing a small number of disulfides into the main chain of polyolefins may enable the copolymers with both degradability and recyclability. In this regard, Johnson et al. reported the redox recycling of deconstructable polystyrenes with low disulfide content [19]. Notably, this involved multiple polymerization and post-modification steps, including the oxidative coupling of thiol-end polymer fragments, to introduce disulfide units into the main chain of polystyrene copolymers (Scheme 1a). The installation of disulfide units plays a crucial role in the closed-loop deconstruction and oxidative repolymerization of polystyrene copolymers while preserving their molar mass and thermomechanical properties. As early as 2014, an alternative way for incorporating disulfide units into linear polyolefin was reported by Emrick and co-workers via ring-opening metathesis copolymerization of disulfide-containing cyclooctene with cyclooctene (Scheme 1b). In their report, the disulfide-containing cyclooctene was required to be synthesized from cyclohexa-1,4-diene through a seven-step pathway [26]. The obtained compound was employed as comonomer with cyclooctene (COE) derivatives for ring-opening metathesis polymerization (ROMP) to provide the disulfide-bond-inserted linear polymers. The introduction of disulfides in the main polymer chain enabled effective degradation upon chemical treatment. It is desirable to develop alternative methods for synthesizing polyolefins with low disulfide content that avoids the need for multiple steps in both monomer and polymer synthesis.

In contrast to the disulfide-containing cyclooctene monomer, diallyl disulfide (DADS) is a naturally occurring compound found in garlic and has shown anti-tumor activity [37,38]. DADS can be easily synthesized by the oxidative dimerization of allyl thiol [39]. Additionally, the metathesis polymerization of cyclic-acyclic monomers for the synthesis of degradable polymers has been rarely reported [40,41]. Herein, we present an alternative strategy for incorporating a cleavable disulfide functional group into the backbone of polyolefins through olefin metathesis copolymerization of DADS with cyclooctene (Scheme 1c). The copolymerization of diallyl disulfide with cyclooctene took place at room temperature using the second-generation Grubbs catalyst, producing copolymers with molecular weight ranging from 5.8 kg·mol^−1^ to 42.8 kg·mol^−1^. This approach offers a convenient means of introducing disulfide units with adjustable content (ranging from 0.7 to 8.5 mol%) into the main polymer chain. The resulting copolymers exhibit favorable crystallinity and demonstrate chemical degradability. Thermal decomposition temperatures of these disulfide-incorporated polymers were much higher than their melting temperatures according to the above thermal data, which indicated that these copolymers possess good thermal processability. Treatment of the copolymer (8.5 mol% disulfide content) with tri-*n*-butylphosphine resulted in a significant reduction in molecular weight from 5.8 kg·mol^−1^ to 1.6 kg·mol^−1^. Successful copolymerization with diallyl disulfide provides a convenient and effective method for obtaining degradable polyolefins.

## 2. Materials and Methods

### 2.1. General Information

All reactions were carried out in an argon-filled glove box or using standard Schlenk techniques under nitrogen. Diallyl disulfide was purchased from Bide Pharmatech Co., Ltd. (Shanghai, China) and redistilled with CaH_2_ and stored in the glove box; cyclooctene was purchased from Beijing InnoChem Science & Technology Co., Ltd. (Beijing, China) and redistilled with CaH_2_ and stored in the glove box. The Grubbs catalysts (G2 and HG2) were purchased from Beijing InnoChem Science & Technology Co., Ltd. and used without further purification, and stored in the glove box. The anhydrous tetrahydrofuran and dichloromethane were obtained through the solvent purification system of Vigor Gas Purification Technologies (Suzhou) Co., Ltd. (Suzhou, China). The anhydrous 1,2-dichloroethane 2-methyltetrahydrofuran was purchased from J&K Scientific Ltd. (Beijing, China) and used without further purification. Tri-*n*-butylphosphine was purchased from Shanghai Titan Technology Co., Ltd. (Shanghai, China) and stored in a glove box. All copolymerizations were performed in a 25-mL Schlenk flask. NMR spectra of polymers were recorded on a Quantum-I Plus (^1^H: 400 MHz, ^13^C: 101 MHz) NMR spectrometer at ambient temperature. Chemical shift values for protons are referenced to the residual proton resonance of chloroform-D (CDCl_3_, δ: 7.26 ppm). Chemical shift values for carbons are referenced to the carbon resonances of chloroform-D (CDCl_3_, δ: 77.16 ppm). Size-exclusion chromatography (SEC) analyses were carried out with an EcoSEC (HLC-8320GPC) equipped with a refractive index (RI) detector by eluting the columns with THF at 1.0 mL/min at 25 °C. Molecular weights were determined using narrow polystyrene standards. Thermogravimetric analyses (TGA) of the resulting polymers were measured on TGA550, and the sample was heated at a rate of 10 °C/min. Differential scanning calorimetry (DSC) measurements of polymers were performed on a DSC 250 analyzer at a heating and cooling rate of 10 °C/min.

### 2.2. Procedure for Homopolymerization of Allyl Disulfide

A 10 mL Schlenk flask with a magnetic stir bar was dried in an oven at 120 °C and then allowed to cool inside a glovebox. Next, the diallyl disulfide and 0.8 mL dry tetrahydrofuran (THF) were added into the Schlenk flask, and the flask was sealed with a rubber plug. Grubbs catalyst was then dissolved in dry THF (0.2 mL) in a vial flask and the solution was added into the Schlenk flask via a syringe. The resulting solution was stirred for 1 h at ambient temperature and then quenched with methyl alcohol. The polymerization mixture was precipitated into 80 mL methyl alcohol, and the solid was obtained by filtration and dried under vacuum at room temperature for 4 h to obtain the required polymer. The polymer structure was determined by NMR spectrogram analysis. The molecular weight and polydispersity were determined by size-exclusion chromatography.

### 2.3. A General Procedure for Copolymerization of Allyl Disulfide and Cyclooctene

A 10 mL Schlenk flask with a magnetic stir bar was dried in an oven at 120 °C and then allowed to cool inside a glovebox. Next, the diallyl disulfide, cyclooctene, and 0.8 mL dry solvent were added into the Schlenk flask, and the flask was sealed with a rubber plug. Grubbs catalyst was then dissolved in corresponding dry solvent (0.2 mL) in a vial flask and the solution was added into the Schlenk flask via a syringe. The resulting solution was stirred for 1 h at ambient temperature and then quenched with methyl alcohol. The polymerization mixture was precipitated into 80 mL methyl alcohol, and the solid was obtained by filtration and dried under vacuum at room temperature for 4 h to obtain the required polymer. The polymer structure was determined by NMR spectrogram analysis. The insertion rate of diallyl disulfide was determined by ^1^H-NMR analysis according to the following equation:

i.r.% = 2g/(2g + c + d) × 100%.

The molecular weight and polydispersity were determined by size-exclusion chromatography.

### 2.4. A General Procedure for Polymer Degradation

A 10 mL Schlenk flask with a magnetic stir bar was placed inside a glovebox. Next, the polymer, tri-*n*-butylphosphine (10 equiv. of the disulfide unit), was added into the Schlenk flask, and the flask was sealed with a rubber plug. The degassed CH_2_Cl_2_ was added into the Schlenk flask via a syringe, and the mixture was stirred at ambient temperature overnight. The solvent was removed under the reduced pressure and the residue was washed with methyl alcohol; the obtained polymer was dried under vacuum at room temperature for 4 h to obtain the required polymer. The molecular weight and polydispersity of the resultant polymer were determined by size-exclusion chromatography.

## 3. Results

### 3.1. Polymerization Experiments

#### 3.1.1. Homopolymerization of Diallyl Disulfide

**Figure 1:**
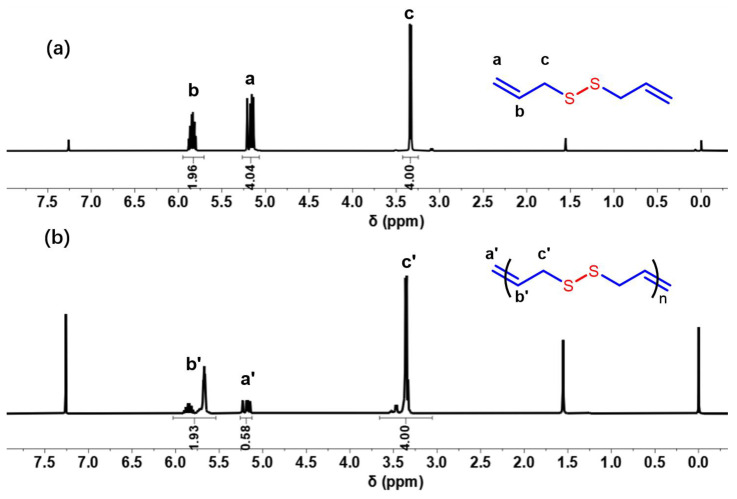
^1^H NMR spectra of diallyl disulfide monomer (**a**) and its homopolymer (**b**).

Initial efforts to homopolymerize diallyl disulfide (DADS) by acyclic diene metathesis polymerization (ADMET) [42,43,44] were tried. When first-generation Grubbs catalyst (G1) was used to proceed with the homopolymerization of diallyl disulfide in tetrahydrofuran (THF) or dichloromethane (DCM), no polymer was obtained when the reaction mixture was precipitated by methanol. When the second-generation Grubbs catalyst (G2) was used at a feed ratio of [DADS]/[Ru] = 500:1 in THF, only oligomers with 13% yield were obtained. Compared with the ^1^H NMR spectrum of the monomer, the integral of the hydrogen signal for terminal olefinic C-H bonds diminished (Figure 1), which indicated the proceeding of ADMET. Quantitative ^1^H NMR analysis suggested that the oligomer consists of about 7 disulfide units, which was also confirmed by size-exclusion chromatography (SEC) analysis (*M*_n_ = 790 g·mol^−1^, Supplementary Information, Figure S22). In fact, disulfide-containing dienes were reported to be reactive in the ring-closing reactions using ruthenium benzylidene catalysts [45]. However, the strong coordination ability of disulfides hinders chain propagation through metathesis polymerization. When diallyl disulfide reacts with the Grubbs catalyst, it leads to the formation of five-membered ruthenium-containing rings [26,45]. These rings prevent other monomers from interacting with the ruthenium center and may ultimately result in catalyst decomposition [46]. Consequently, similar to the homopolymerization of diallyl disulfide, the reported olefin metathesis homopolymerization of disulfide-containing monomers only yields low molar mass oligomers [26,47].

#### 3.1.2. Copolymerization of Diallyl Disulfide and Cyclooctene

Based on the experimental results of DADS homopolymerization, G2 was first tested in the copolymerization of diallyl disulfide and cyclooctene at a feed ratio of [COE]/[DADS]/[Ru] = 1000:50:1 in tetrahydrofuran. A copolymer (0.318 g) was isolated after precipitation with methanol. In the ^1^H NMR of the obtained copolymer, characteristic signals at δ = 3.30 ppm (d) corresponding to the allylic C-H were observed. The incorporation ratio of disulfide units was determined as 4.1 mol%. The molecular weight and polydispersity were determined by SEC as *M*_n_ = 7.9 kg·mol^−1^, and *M*_w_/*M*_n_ = 1.8, respectively (Table 1, entry 1). Compared to the G2 catalyst, the Hoveyda–Grubbs catalyst (HG2) is equipped with a larger sterically hindered isopropoxy group and possesses a more stable structure [48]. When used for the copolymerization process, it leads to a higher yield of copolymer (Table 1, entry 2). The third-generation Grubbs catalyst (G3) produced copolymers with slightly decreased molecular weight but similar disulfide incorporation ratios (Table 1, entry 3). It has been reported that the strong coordination ability of disulfides hinders chain propagation through metathesis polymerization due to the formation of five-membered ruthenium-containing rings. The decrease in copolymer molecular weight using HG2 and G3 might be explained by the easier formation of five-membered ruthenium-containing rings with facile pyridine ligand dissociation. Thereafter, other solvents such as dichloromethane (DCM), dichloroethane (DCE), and 2-methyltetrahydrofuran (2-Me-THF) were investigated in the presence of G2 (Table 1, entries 4–6). The copolymerization in DCM and DCE gave slightly increased disulfide incorporation ratios, whereas the 2-Me-THF produced a copolymer with decreased molecular weight and broadened polydispersity. DCM was chosen as the solvent for further exploration.

**Figure 2:**
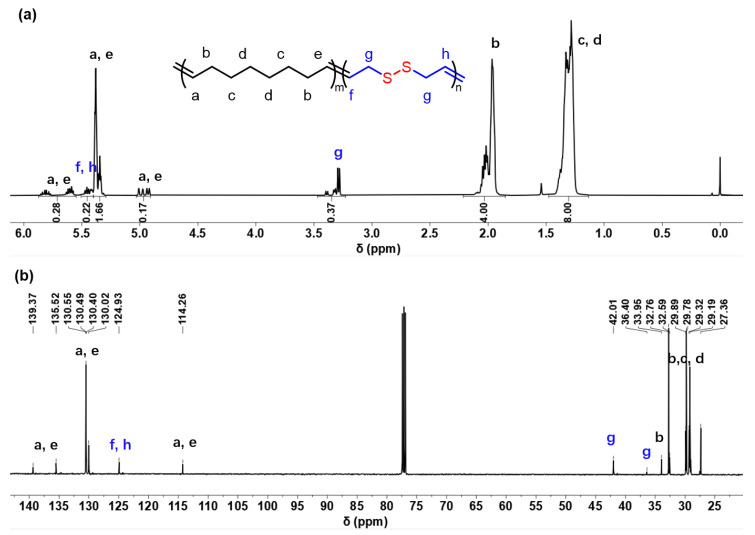
^1^H NMR (**a**) and ^13^C NMR (**b**) spectra of polymer obtained in Table 1, Entry 10.

In the subsequent step, various feed ratios of the monomers were examined to adjust the incorporation ratios of disulfide units. Reducing the amount of DADS resulted in lower disulfide incorporation ratios but higher molecular weights (Table 1, entries 7 and 8). Conversely, increasing the feed ratio of DADS had a positive impact on the insertion of disulfide units but led to a slight decrease in molecular weights and dispersity (Table 1, entries 9 and 10). The highest disulfide incorporation ratio was achieved with a COE/DADS feed ratio of 10:1, yielding a copolymer with 8.5% disulfide units (Table 1, Entry 10, *M*_n_ = 5.8 kg·mol^−1^, approximately 5 disulfide units per chain). As illustrated in Figure 2, the distinctive doublet corresponding to the alpha position of the disulfide unit at δ = 3.30 ppm, along with the signals for polycyclooctene, was observed in the ^1^H NMR spectrum of this copolymer. Furthermore, the olefinic proton signals (f and h) adjacent to the disulfide groups were positioned at δ = 5.45 ppm based on ^1^H-^1^H COSY-NMR analysis (Supplementary Information, Figure S16). In the ^13^C NMR, the chemical shifts corresponding to the disulfide units were found at δ = 42.0, 36.4 ppm (alpha carbon), and δ = 124.9 ppm (beta olefin carbon). The catalyst loading had minimal impact on the disulfide incorporation ratios or molecular weights, but it significantly affected the polymerization rate (Table 1, entries 11 and 12).

### 3.2. Thermal Properties of Copolymers

**Figure 3:**
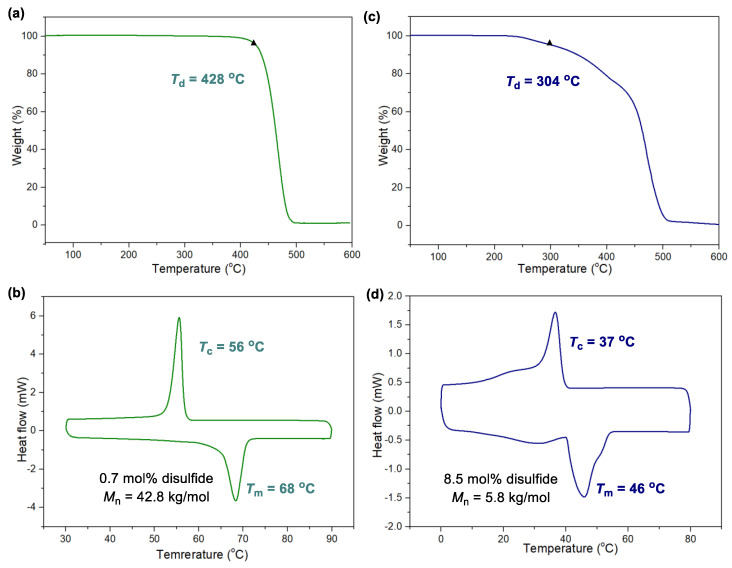
Thermal properties of the obtained copolymers. (**a**) TGA curve of the copolymer in Table 1, entry 7. (**b**) DSC trace of the copolymer in Table 1, entry 7. (**c**) TGA curve of the copolymer in Table 1, entry 10. (**d**) DSC trace of the copolymer in Table 1, entry 10.

Efforts were then paid to evaluate the thermal properties of the copolymers. First, thermogravimetric analysis (TGA) of the copolymer with 0.7 mol% disulfide content (Table 1, entry 7, *M*_n_ = 42.8 kg·mol^−1^) and the copolymer with 8.5 mol% disulfide content (Table 1, entry 10, *M*_n_ = 5.8 kg·mol^−1^) were both carried out. As shown in Figure 3, the copolymer with low disulfide content showed compatible thermostability (*T*_d_ = 428 °C) with commercial polyethylene (PE) materials, whereas a lower decomposition temperature (*T*_d_ = 304 °C) was observed in the case of the copolymer with 8.5 mol% disulfide content. A sharp endothermic peak at *T*_m_ = 68 °C and a clear exothermic peak at *T*_c_ = 56 °C were detected for the copolymer with 0.7 mol% disulfide content (Figure 3b). Meanwhile, the copolymer with higher disulfide incorporation (8.5 mol%) exhibited lower melting and crystallization temperatures at *T*_m_ = 46 °C and *T*_c_ = 37 °C, respectively. Thermal decomposition temperatures of these disulfide-incorporated polymers were much higher than their melting temperatures according to the above thermal data, which indicated that these copolymers possess good thermal processability.

### 3.3. Polymer Degradation

**Figure 4:**
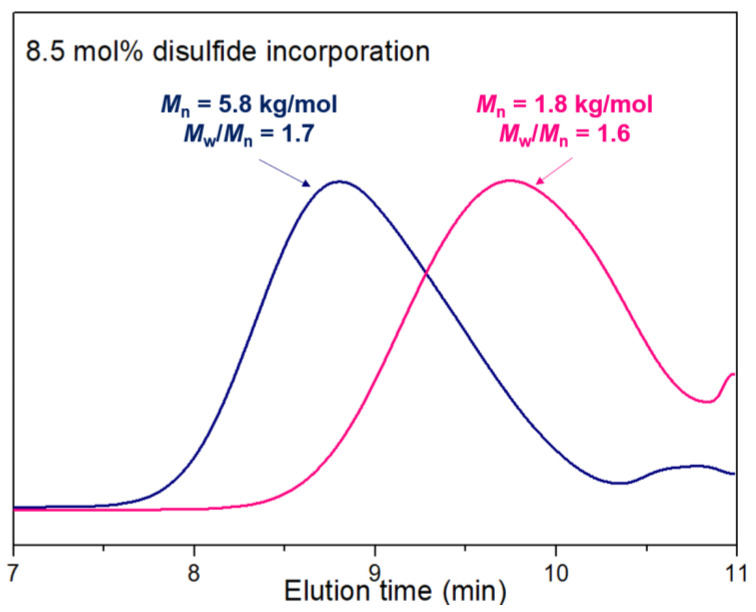
SEC trace of the copolymer in Table 1, entry 10 before (blue line) and after (pink line) treatment with tri-*n*-butylphosphine.

Compared with traditional polyolefin materials, the insertion of disulfide units into the main chain renders polymers degradable. The degradability of the copolymer in Table 1, entry 10 with 8.5 mol% disulfide content was evaluated. The disulfide bond was reported to cleave and transform into the corresponding thiol in the presence of tri-substituted phosphine [49]. The one sulfur atom of disulfide would be attacked by lone pair electrons of the phosphine, the disulfide bond was broken, and the generated sulfur anion could be captured by protons to yield one thiol product. Meanwhile, the P-S cation species would be oxidated by water to produce another thiol and phosphine oxide [50]. As a result, the copolymer was treated with 10 equiv of *n*-Bu_3_P, then precipitated in methanol to obtain the degradated product. GPC analysis showed molecular weight of the copolymer decreased significantly from 5.8 kg·mol^−1^ to 1.6 kg·mol^−1^ (Figure 4). These experiments revealed that the insertion of low-content disulfide units into the polyolefin backbone would endow it with biodegradability but retain its thermal properties.

## 4. Conclusions

In conclusion, we have presented a novel approach for incorporating the disulfide functional group into the backbone of polyolefins using commercially available diallyl disulfide as a comonomer through metathesis copolymerization in spite of the homopolymerization of diallyl disulfide only resulting in an oligomer with the molecular weight of 790 g·mol^−1^. The copolymerization of diallyl disulfide with cyclooctene occurred using the second-generation Grubbs catalyst under mild conditions, allowing for the synthesis of copolymers with adjustable disulfide content (ranging from 0.7 to 8.5 mol%), and the molecular weight of the obtained copolymers ranged from 5.8 kg·mol^−1^ to 42.8 kg·mol^−1^. Thermal decomposition temperatures of these disulfide-incorporated polymers were much higher than their melting temperatures according to the above thermal data, which indicated that these copolymers possess good thermal processability. A significant reduction in molecular weight was observed upon treatment of the copolymer (8.5 mol% disulfide content) with tri-*n*-butylphosphine (from 5.8 kg·mol^−1^ to 1.6 kg·mol^−1^). The successful copolymerization with diallyl disulfide offers a convenient and effective pathway to obtain degradable polyolefins.

## Figures and Tables

**Table 1:** Copolymerization of cyclooctene and diallyl disulfide *^a^*.

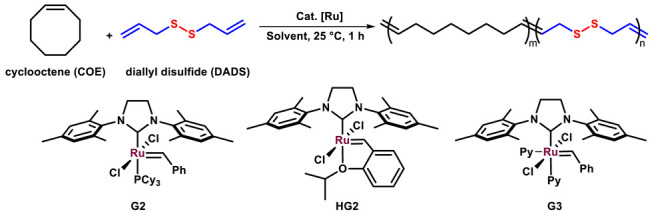							
Entry	Cat. [Ru]	[COE]:[DADS]:[Ru]	Solvent	Yield (mg)	i.r. *^b^* (%)	*M*_n_ *^c^* (kg·mol^−1^)	*M*_w_/*M*_n_*^c^*
1	G2	1000:50:1	THF	318.1	4.1	7.9	1.82
2	HG2	1000:50:1	THF	365.1	4.1	6.6	1.75
3	G3	1000:50:1	THF	325.2	4.3	6.5	1.80
4	G2	1000:50:1	DCM	352.7	4.5	8.0	1.82
5	G2	1000:50:1	DCE	337.1	4.3	8.3	1.93
6	G2	1000:50:1	2-Me-THF	362.7	4.5	6.5	2.00
7	G2	1000:10:1	DCM	401.2	0.7	42.8	1.79
8	G2	1000:25:1	DCM	392.9	2.0	13.9	2.22
9	G2	1000:75:1	DCM	274.1	6.3	5.9	1.74
10	G2	1000:100:1	DCM	221.2	8.5	5.8	1.72
11 *^d^*	G2	5000:250:1	DCM	356.3	4.5	8.5	1.78
12	G2	100:5:1	DCM	362.6	4.5	9.1	1.99

*^a^* Reaction conditions: cyclooctene (4.0 mmol), diallyl disulfide, and Grubbs catalyst in 1.0 mL solvent, stirred at 25 °C for 1 h. ***^b^*** Incorporation ratio (i.r.) of diallyl disulfide in mol% determined by ^1^H-NMR analysis. ***^c^*** Molecular weights and polydispersity determined by size-exclusion chromatography in THF using polystyrene standards. ***^d^*** Reaction for 5 h.

## Data Availability

The data presented in this study are available in this article.

## Supplementary Materials

The following supporting information can be downloaded at: https://www.mdpi.com/article/10.3390/polym15143101/s1, Figures S1–S17: Copies of NMR spectra; Figures S18 and S19: Copies of TGA curves; Figures S20 and S21: Copies of DSC traces; Figures S22–S35: Copies of SEC charts.
