# Supplementary material for: Synthesis of Degradable Polyolefins Bearing Disulfide Units via Metathesis Copolymerization
Source: Polymers (Basel). 2023 Jul 20;15(14):3101. doi: 10.3390/polym15143101 (PMC10384691; doi:10.3390/polym15143101)
Supplement: Supplementary file 1 [file polymers-15-03101-s001.zip › polymers-2497166-supplementary.pdf]

Supplementary Materials

Synthesis of Degradable Polyolefins Bearing Disulfide Units via Metathesis Copolymerization

Yu Xia [1,2,†], Fulin Zhou [2,†], Wenyan Hao [1,*] and Shan Tang [2,*]

[1] College of Chemistry and Chemical Engineering, Jiangxi Normal University, Nanchang 330022, China
[2] Frontiers Science Center for Transformative Molecules, School of Chemistry and Chemical Engineering, Shanghai Jiao Tong University, Shanghai 200240, China
* Correspondence: wenyanhao@jxnu.edu.cn (W.H.); tang.shan@sjtu.edu.cn (S.T.)
† These authors contributed equally to this work.

Contents

1. Copies of NMR spectra 2

2. Copies of TGA curves 11

3. Copies of DSC Trace 12

4. Copies of SEC Charts 13

1. Copies of NMR spectra

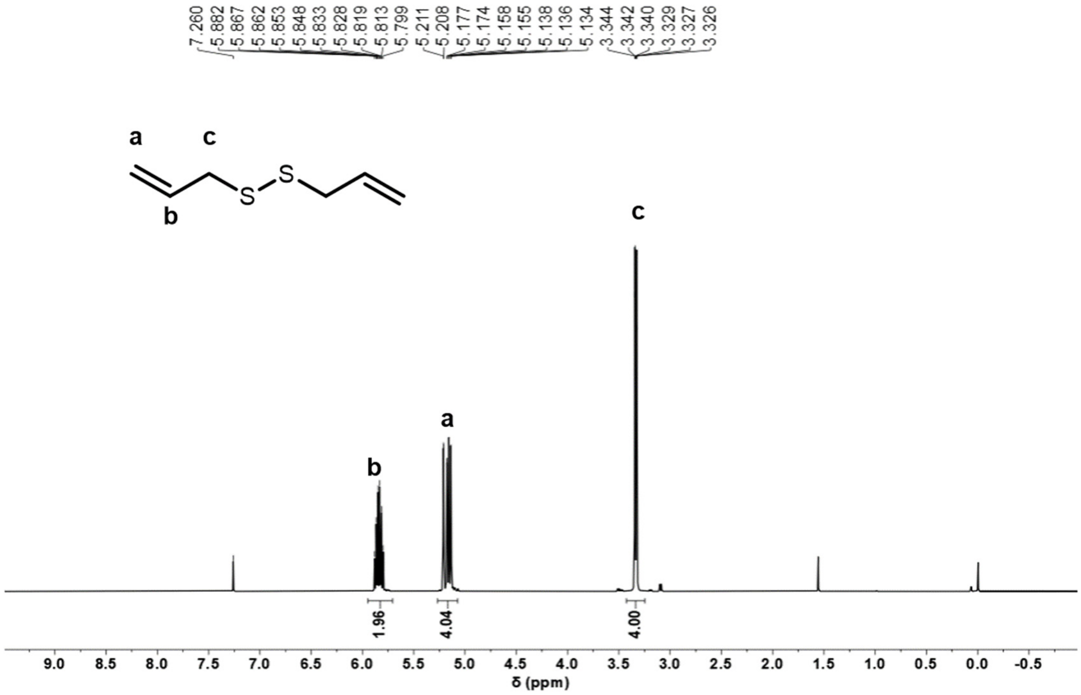

Figure S1. ^{1}H NMR spectrum of diallyl disulfide.

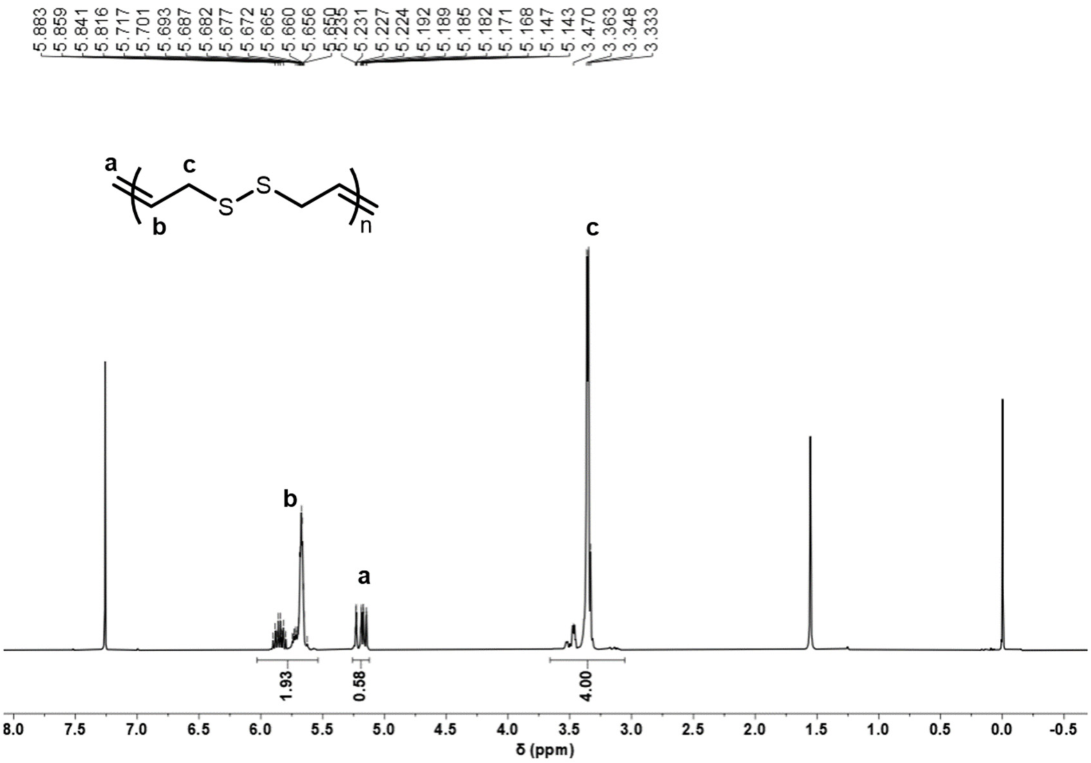

Figure S2. ^{1}H NMR spectrum of homopolymer of diallyl disulfide.

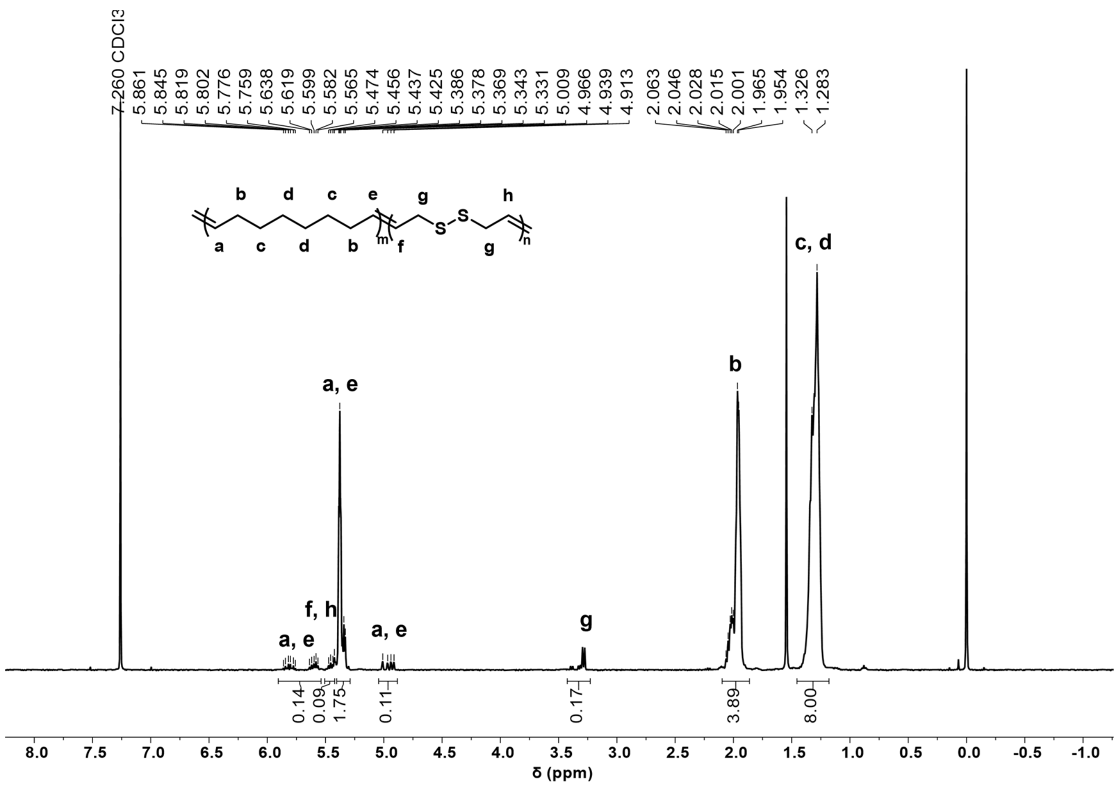

Figure S3. ^{1}H NMR spectrum of polymer obtained in Table 1, Entry 1.

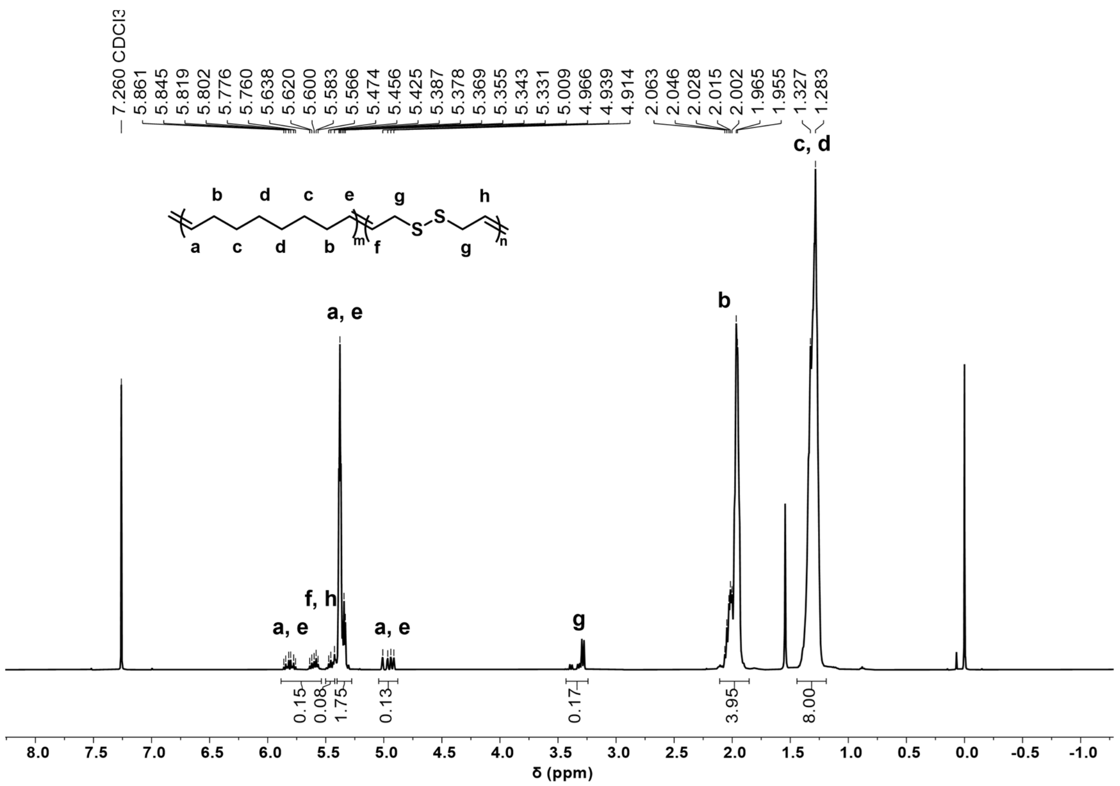

Figure S4. ^{1}H NMR spectrum of polymer obtained in Table 1, Entry 2.

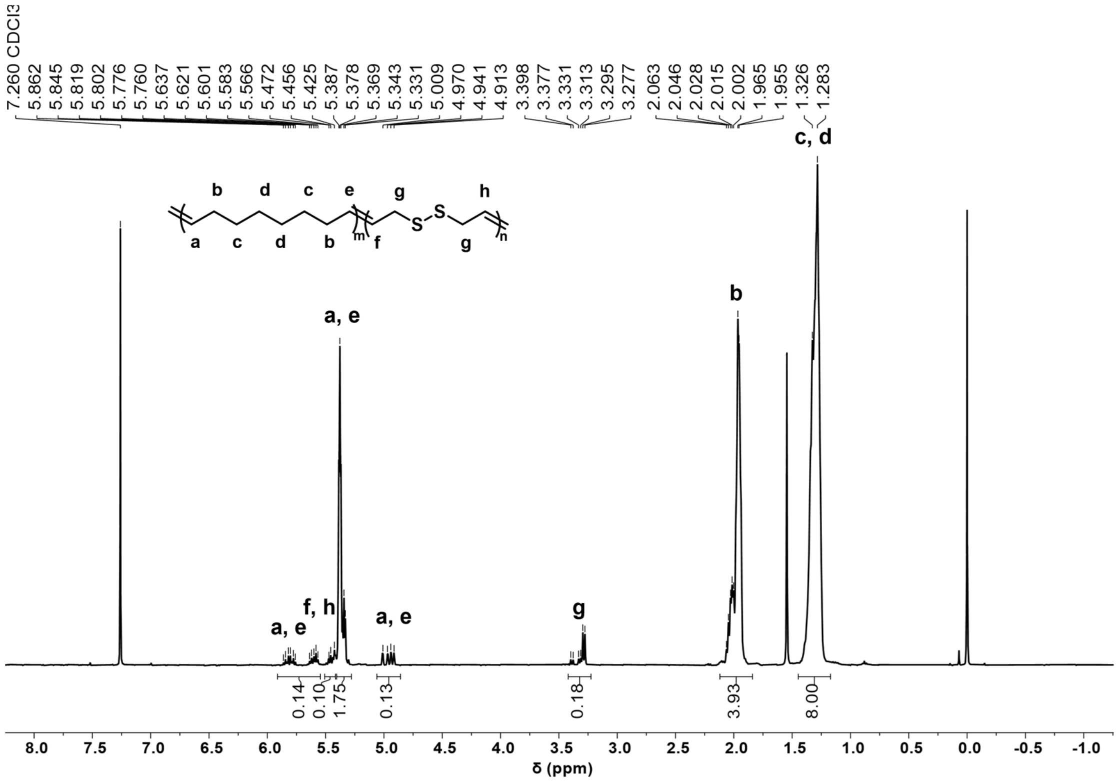

Figure S5. ^{1}H NMR spectrum of polymer obtained in Table 1, Entry 3.

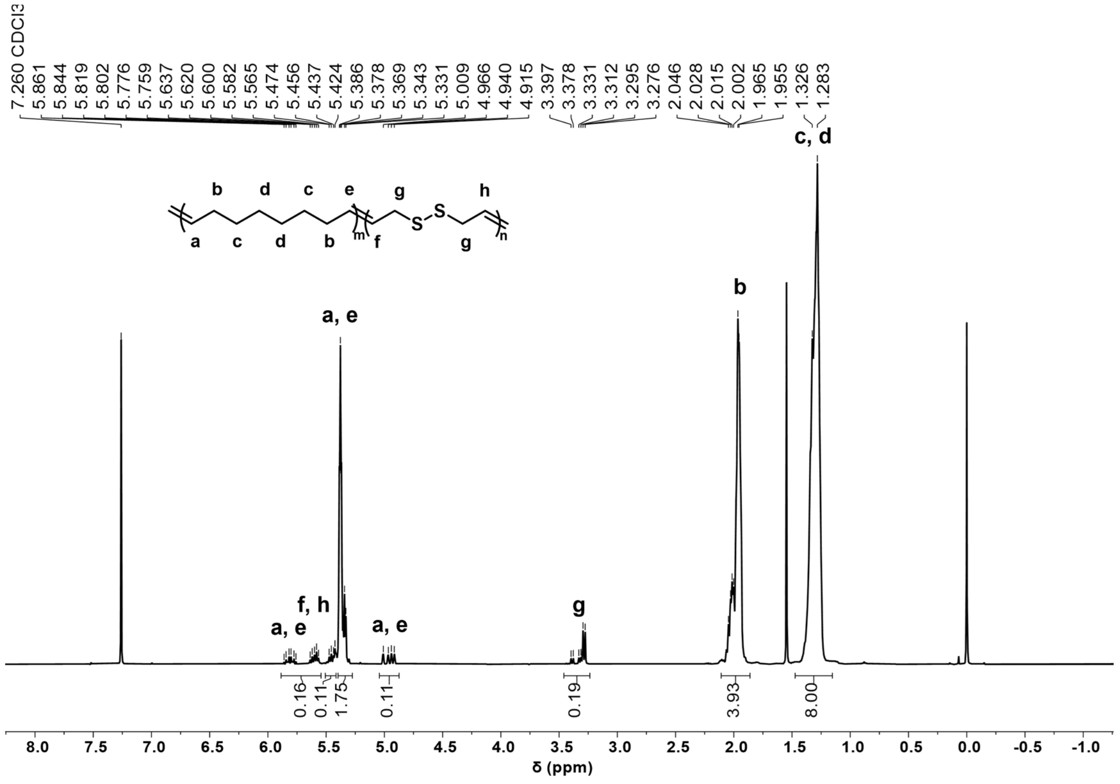

Figure S6. ^{1}H NMR spectrum of polymer obtained in Table 1, Entry 4.

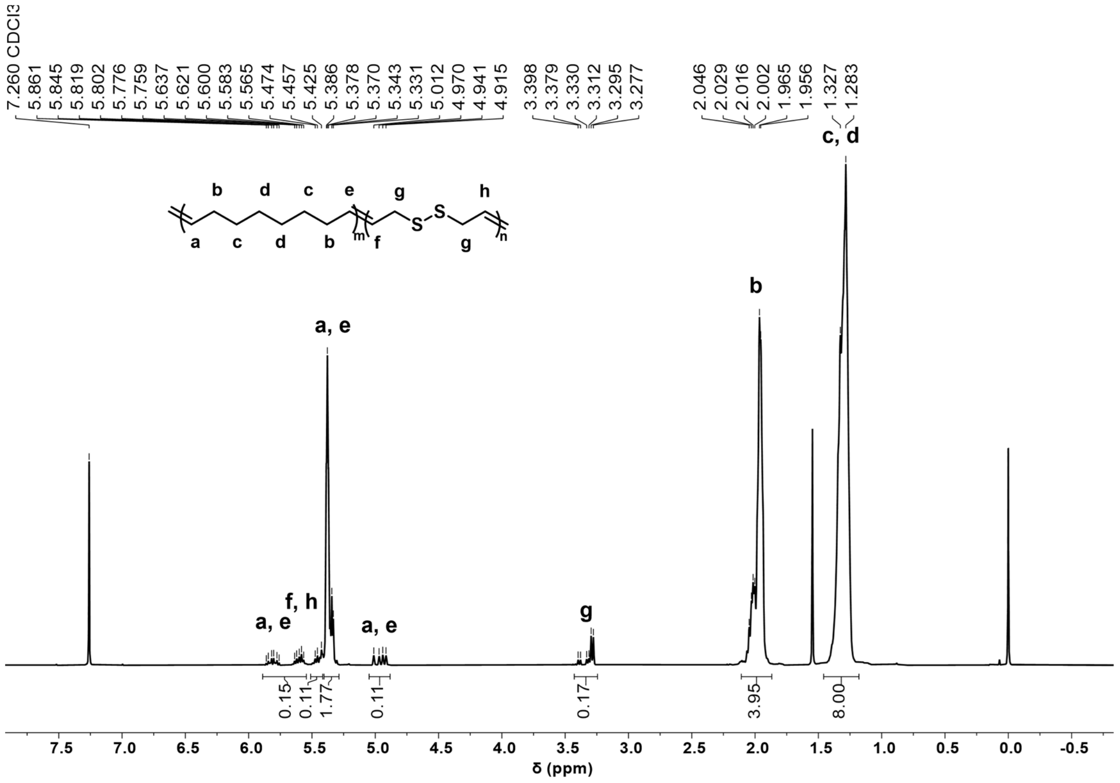

Figure S7. ^{1}H NMR spectrum of polymer obtained in Table 1, Entry 5.

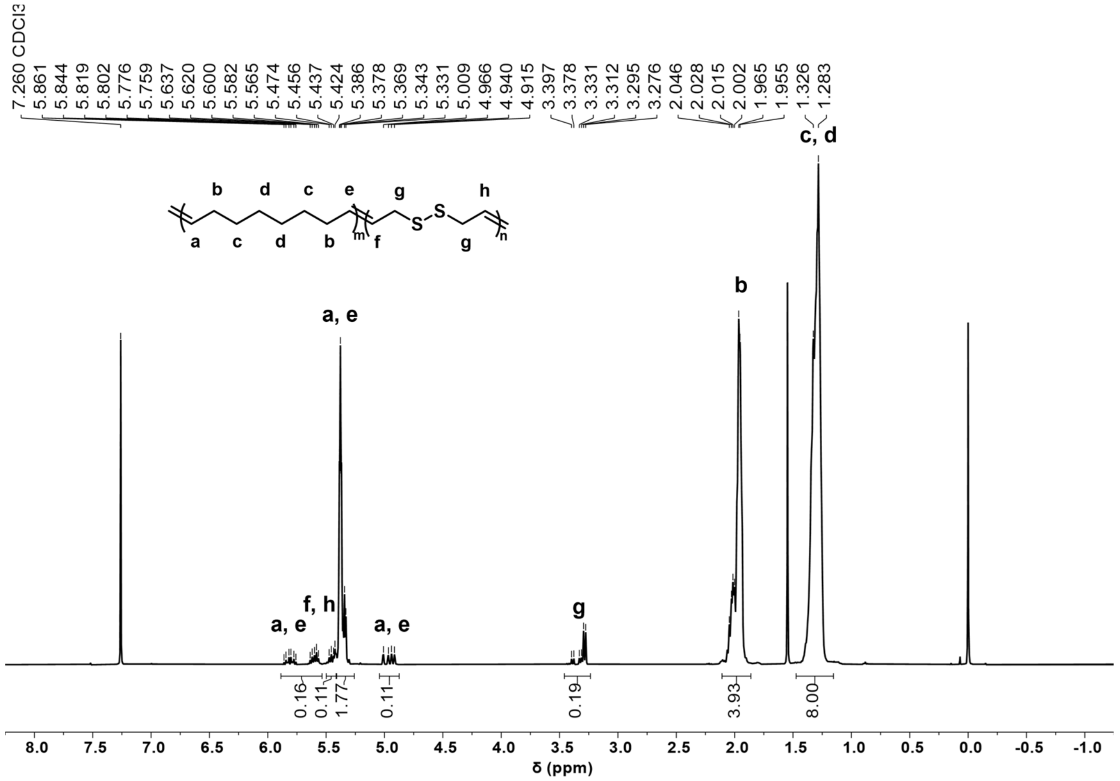

Figure S8. ^{1}H NMR spectrum of polymer obtained in Table 1, Entry 6.

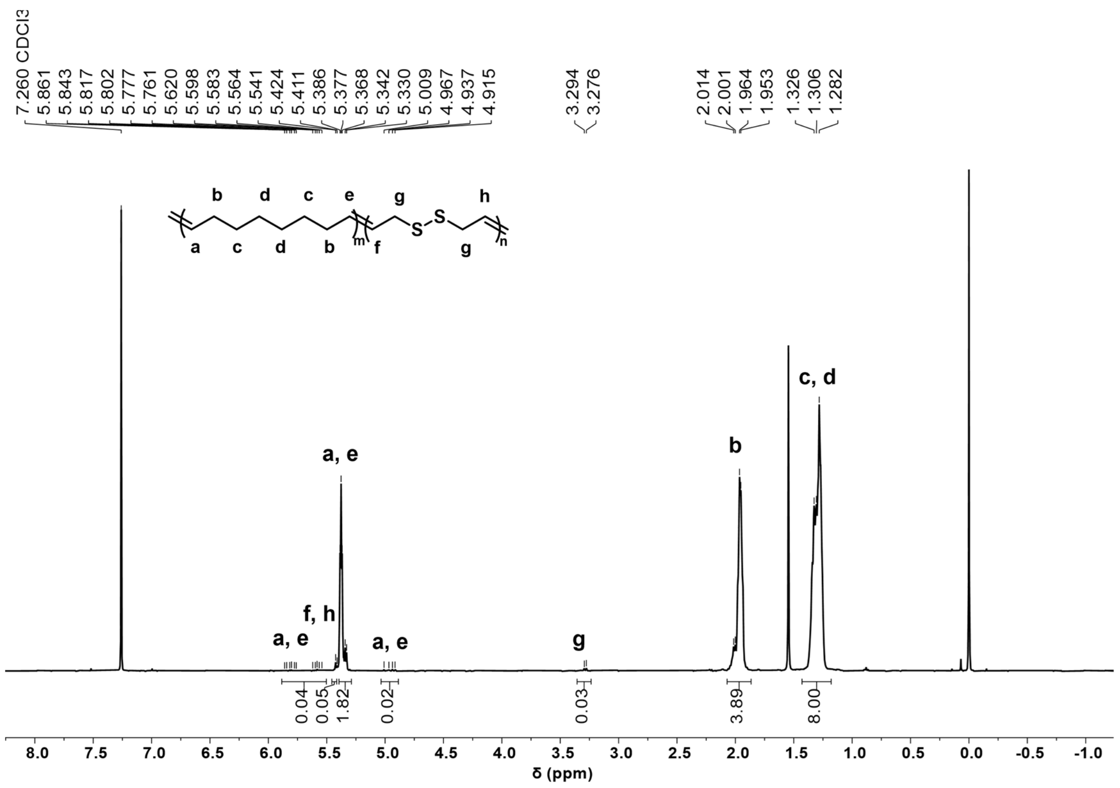

Figure S9. ^{1}H NMR spectrum of polymer obtained in Table 1, Entry 7.

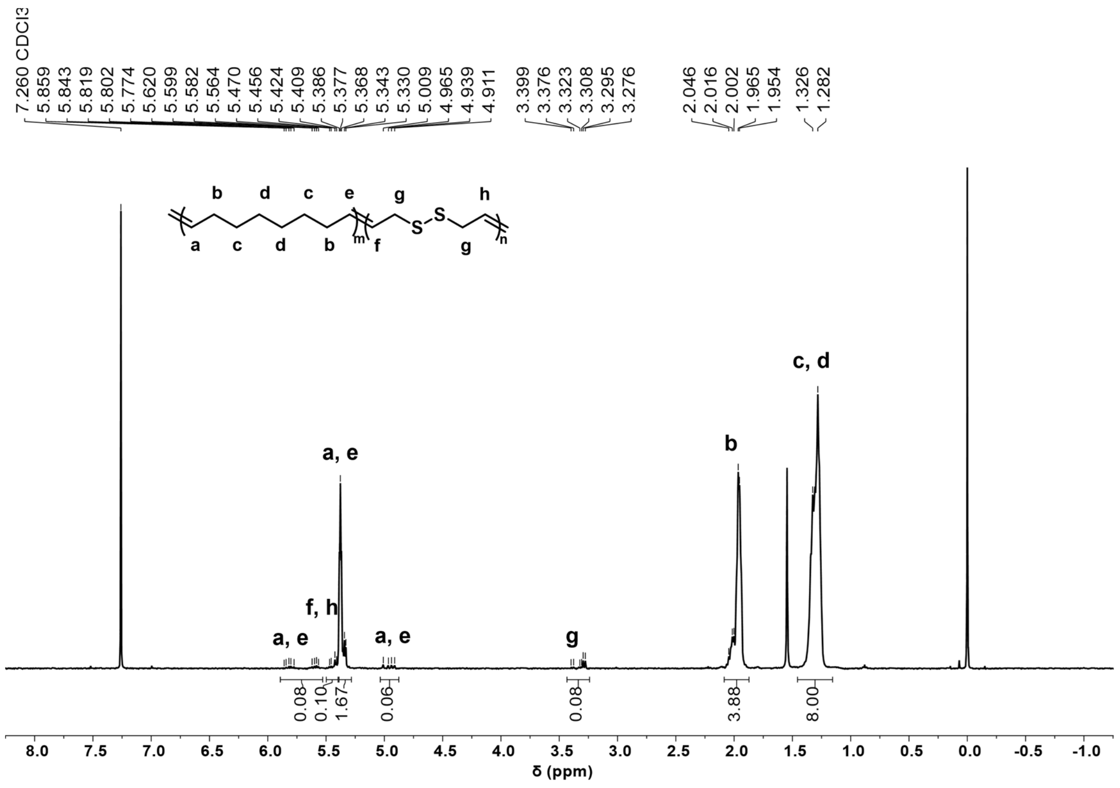

Figure S10. ^{1}H NMR spectrum of polymer obtained in Table 1, Entry 8.

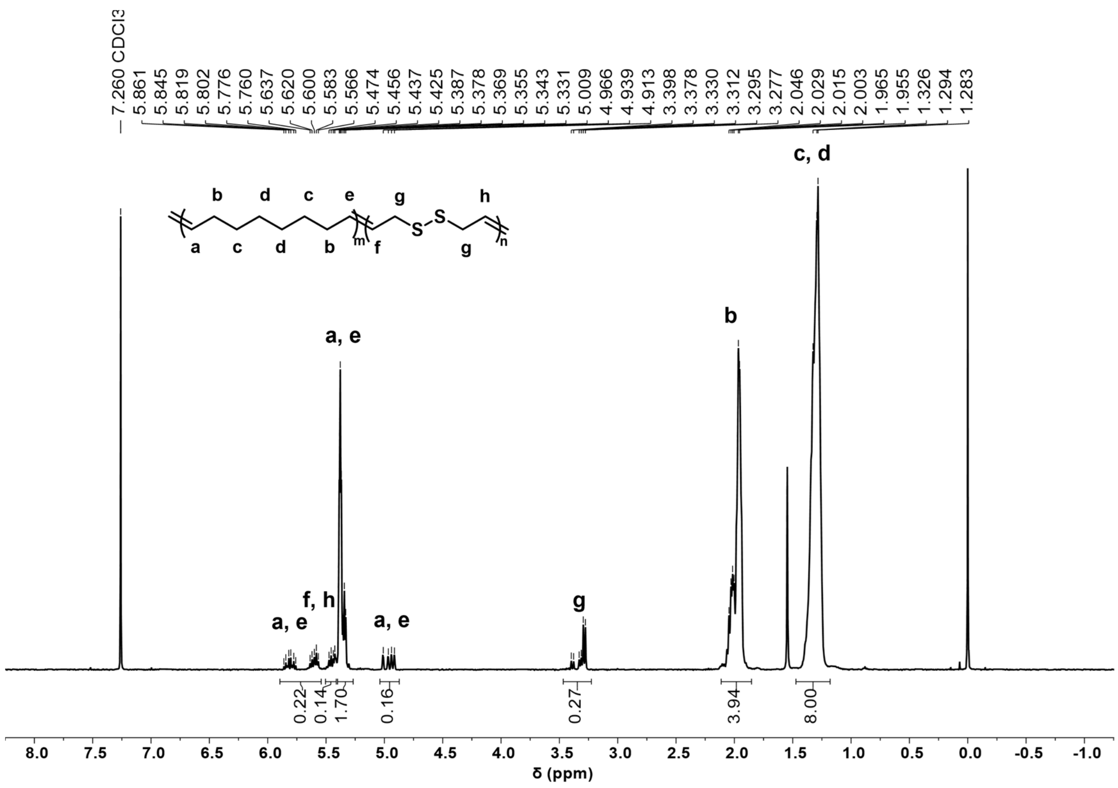

Figure S11. ^{1}H NMR spectrum of polymer obtained in Table 1, Entry 9.

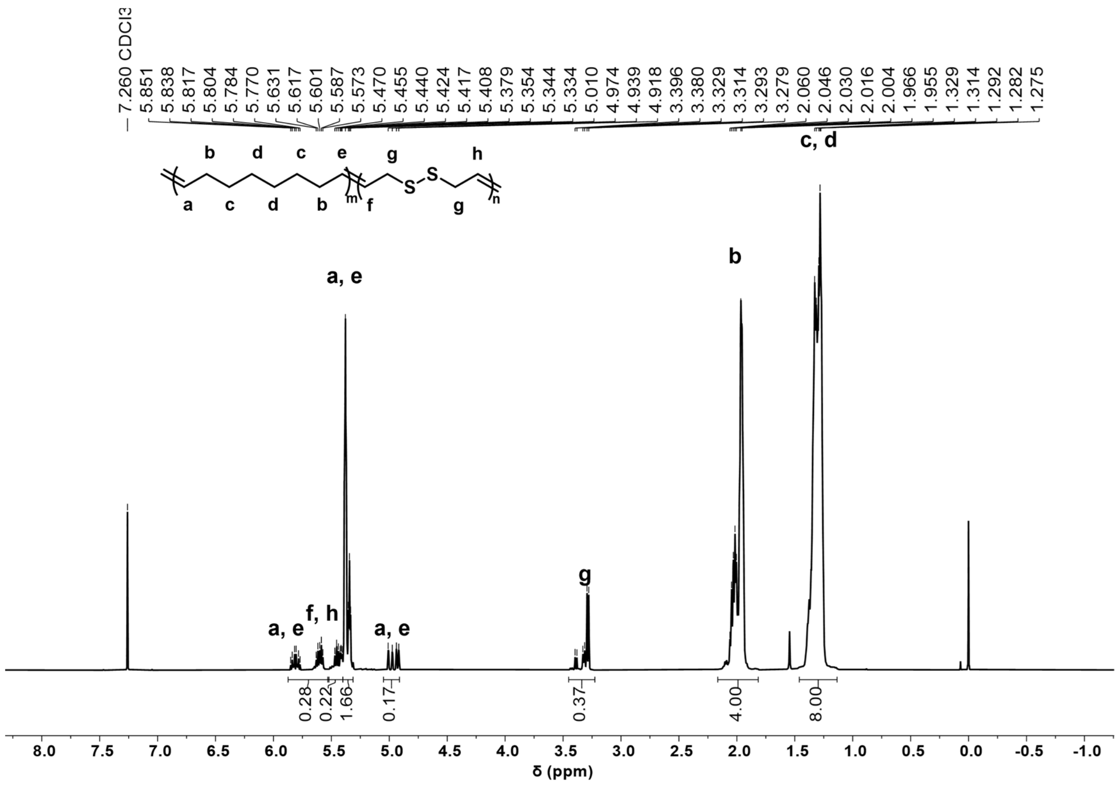

Figure S12. ^{1}H NMR spectrum of polymer obtained in Table 1, Entry 10.

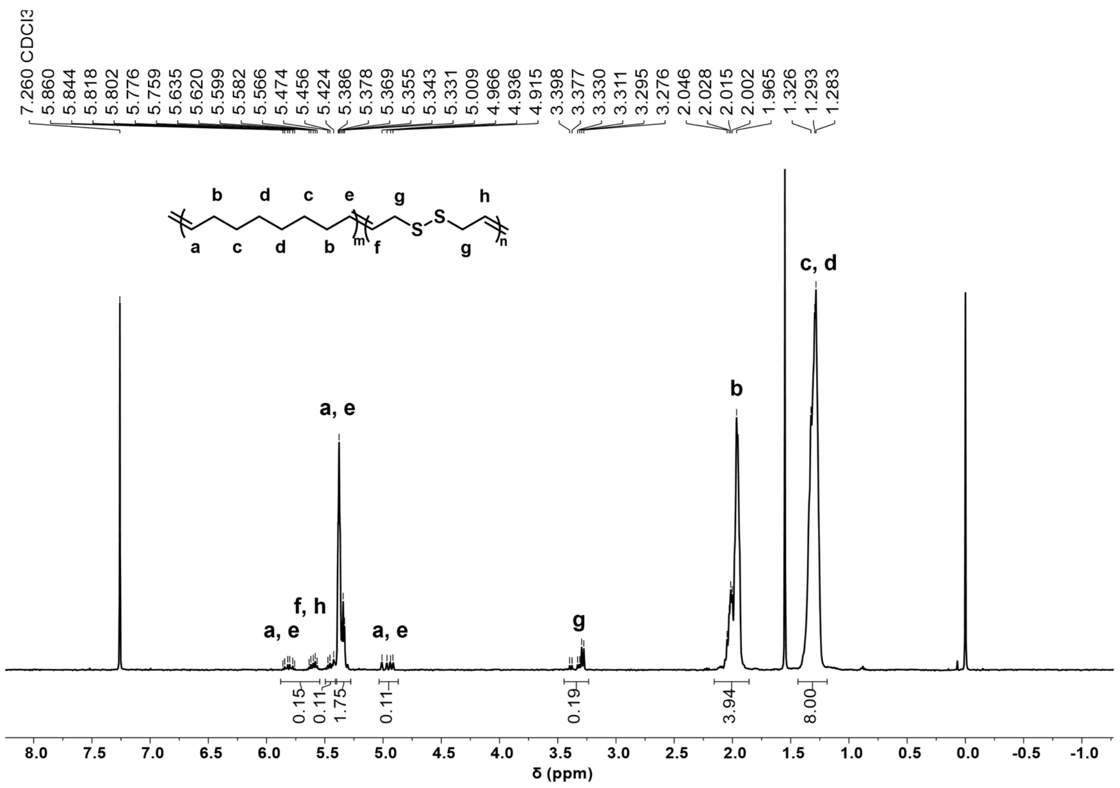

Figure S13. ^{1}H NMR spectrum of polymer obtained in Table 1, Entry 11.

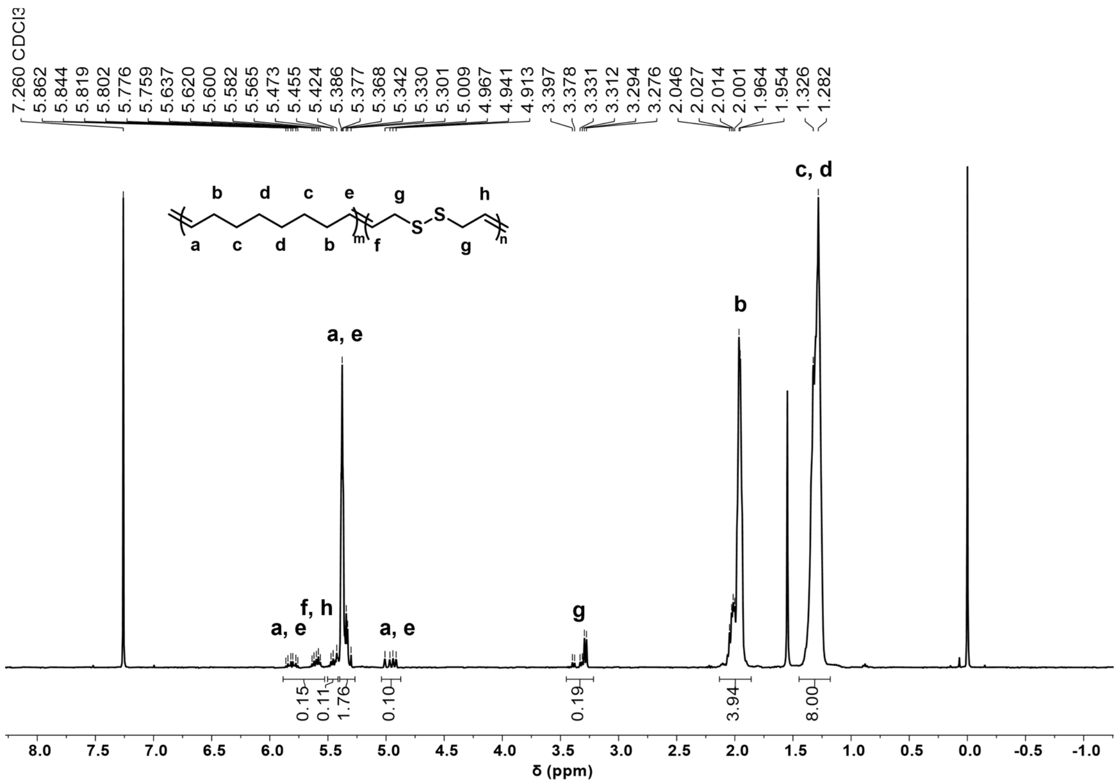

Figure S14. ^{1}H NMR spectrum of polymer obtained in Table 1, Entry 12.

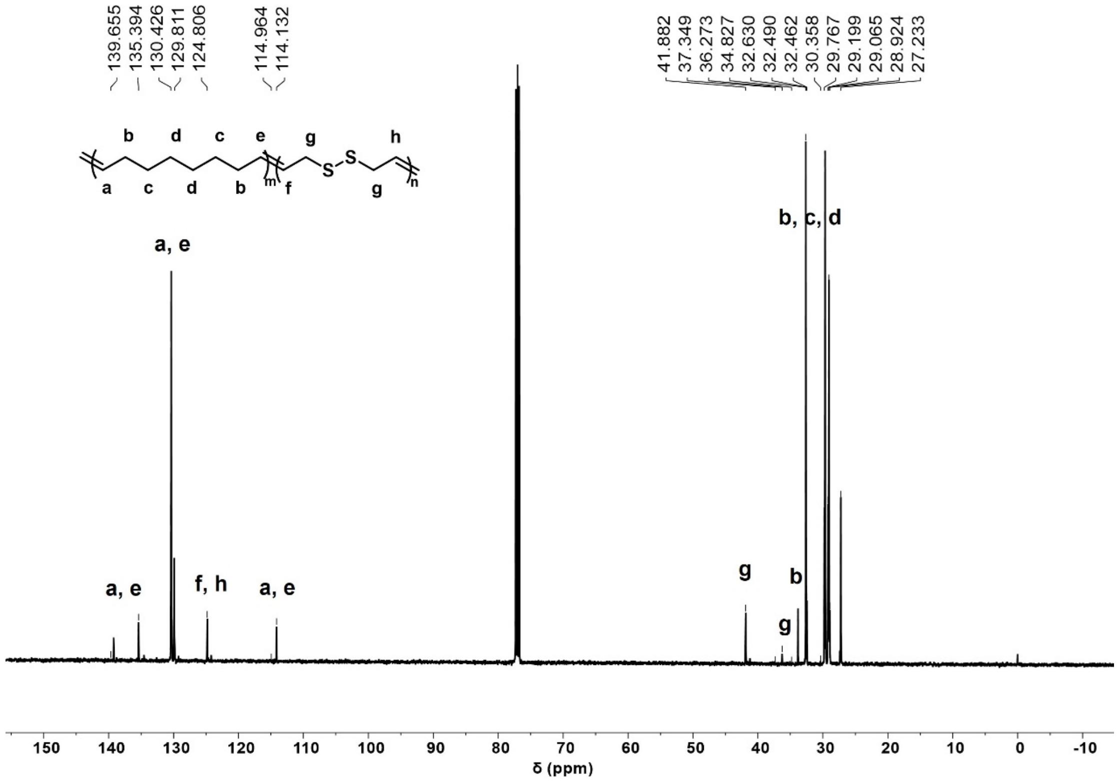

Figure S15. ^{13}C NMR spectrum of polymer obtained in Table 1, Entry 10.

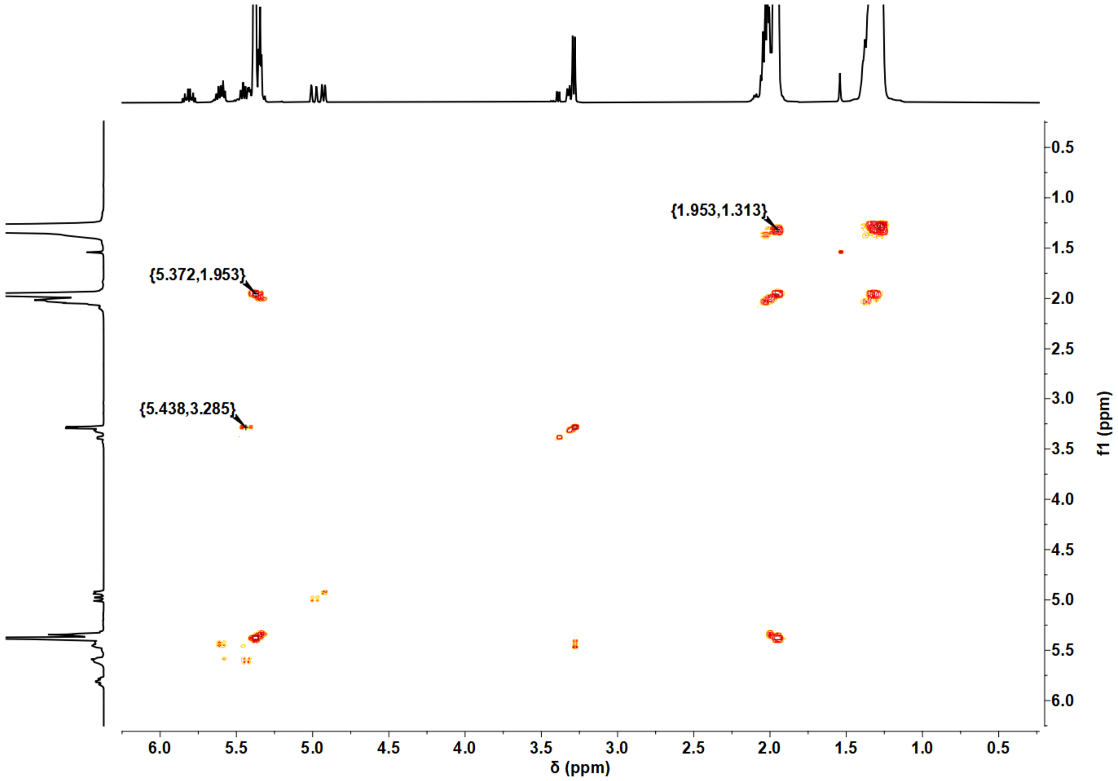

Figure S16. 2D ^{1}H-^{1}H COSY-NMR spectrum of polymer obtained in Table 1, Entry 10.

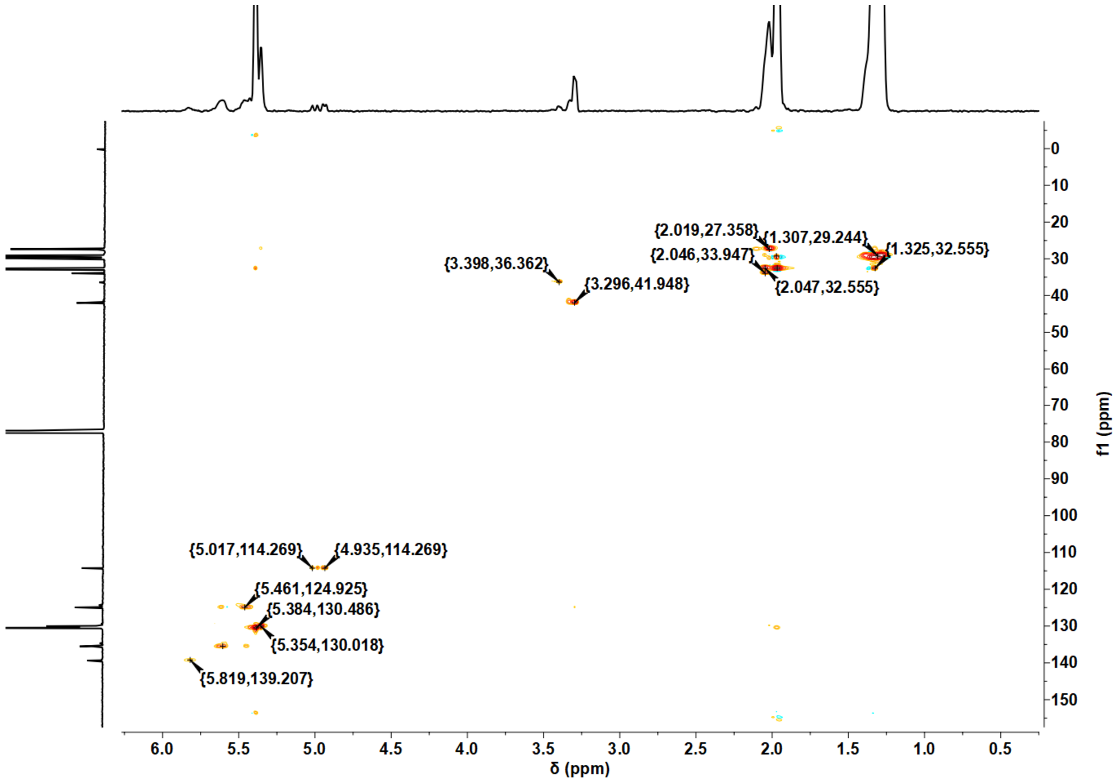

Figure S17. 2D ^{1}H-^{13}C HSQC NMR spectrum of polymer obtained in Table 1, Entry 10.

2. Copies of TGA curves

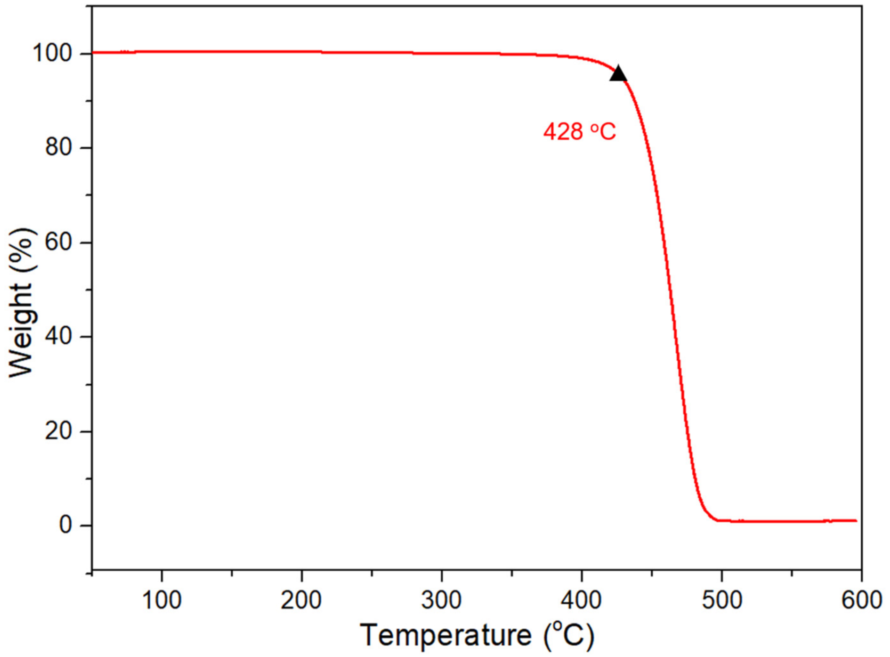

Figure S18. TGA curve of polymer obtained in Table 1, Entry 7.

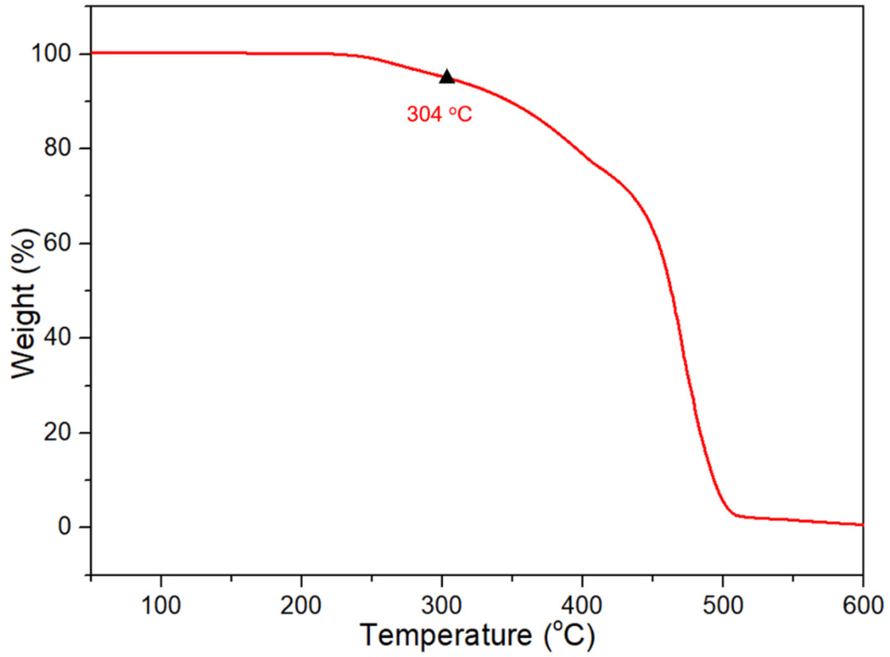

Figure S19. TGA curve of polymer obtained in Table 1, Entry 10.

3. Copies of DSC Trace

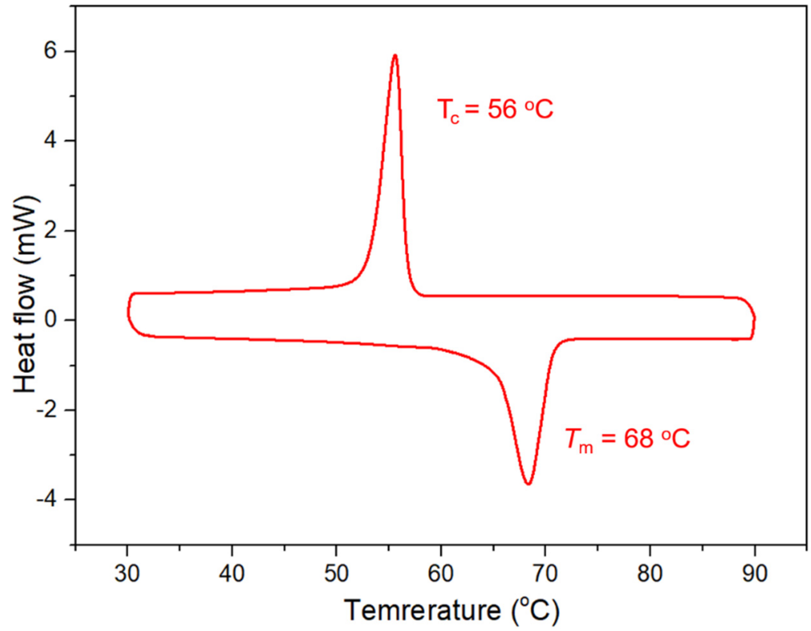

Figure S20. DSC trace of polymer obtained in Table 1, Entry 7.

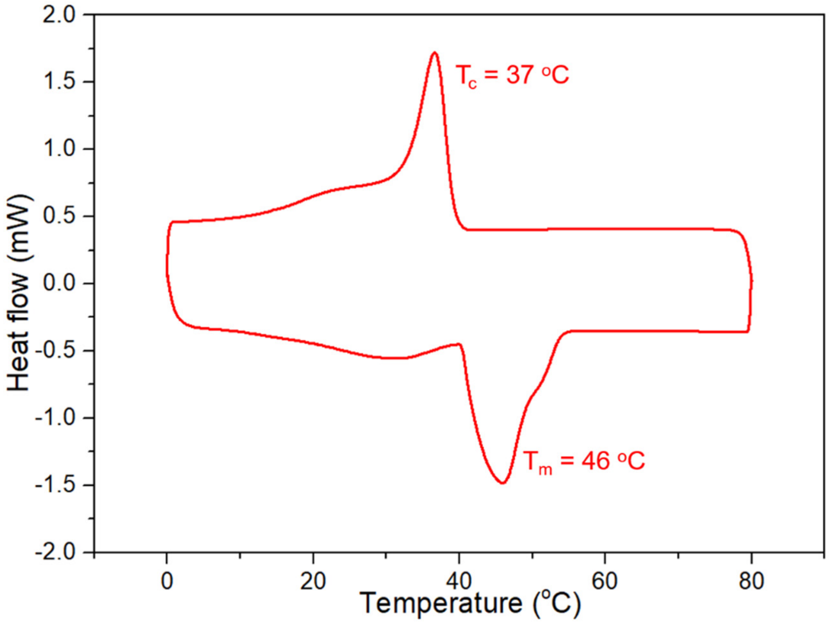

Figure S21. DSC trace of polymer obtained in Table 1, Entry10.

4. Copies of SEC Charts

GPC TEST REPORT

Header

Title		Data acquisition date and time	2022/08/17 15:16:20
Sample name	ZFL-1-53-1	Calculation date and time	2022/08/17 20:13:27
Database name	22-0817.chd	Acquisition time [min]	0.000 - 28.000
Data name	RSLT0007	Sampling interval [msec]	100
Method name	PS-20220628	Cup number	7
Channel	RI	Calculation type	Molecular Weight

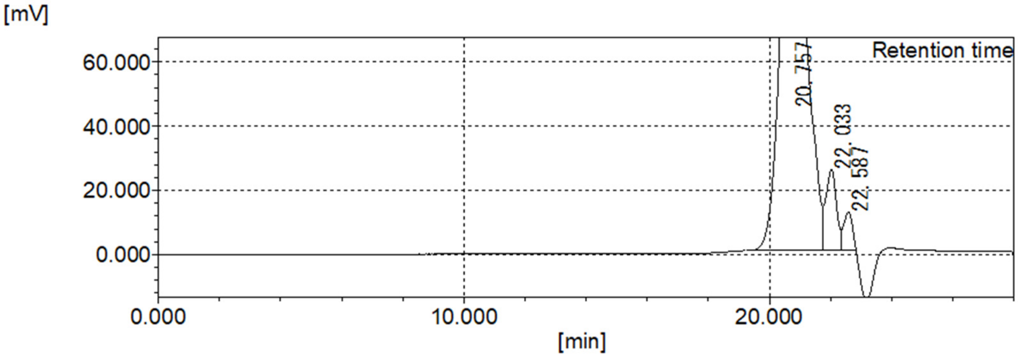

Result of molecular weight calculation (RI)

Peak 1 Base Peak

	[min]	[mV]	[mol]		
Peak start	19.507	1.513	5,626	Mn	790
Peak top	20.757	176.375	1,029	Mw	1,095
Peak end	21.750	15.110	180	Mz	1,429
				Mz+1	1,794
				Mv	1,095
Height [mV]			174.889	Mp	1,057
Area [mV*sec]			8530.908	Mz/Mw	1.305
Height% [%]			82.553	Mw/Mn	1.386
[eta]			1095.20093	Mz+1/Mw	1.638

Figure S22. SEC chart of the homopolymer of diallyl disulfide (Mn = 790 g·mol^{-1}, Mw/Mn = 1.39)

Header

Title		Data acquisition date and time	2023/03/14 21:03:17
Sample name	GRH-YP202312717-XY-69-1	Calculation date and time	2023/03/23 10:22:28
Database name	2023-03-14.chd	Acquisition time [min]	0.000 - 20.000
Data name	RSLT4535	Sampling interval [msec]	100
Method name	PS-20230320	Cup number	9
Channel	RI	Calculation type	Molecular Weight

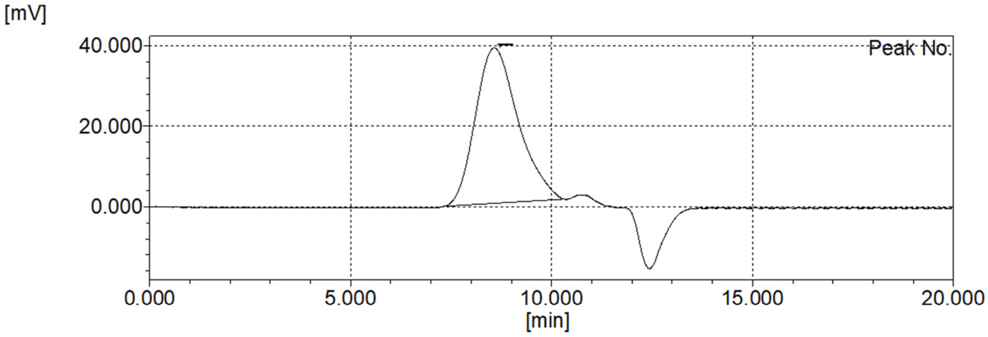

Result of molecular weight calculation (RI)

Peak 1 Base Peak

	[min]	[mV]	[mol]		
Peak start	7.312	0.094	95,185	Mn	7,947
Peak top	8.572	39.603	13,343	Mw	14,513
Peak end	10.305	1.887	894	Mz	22,677
				Mz+1	31,364
				Mv	14,513
Height [mV]			38.754	Mp	13,344
Area [mV*sec]			2857.613	Mz/Mw	1.563
Area% [%]			100.000	Mw/Mn	1.826
[eta]			14512.61128	Mz+1/Mw	2.161

Figure S23. SEC chart of the polymer obtained in Table 1, entry 1. (Mn = 7.9 kg·mol^{-1}, Mw/Mn = 1.83)

Header

Title		Data acquisition date and time	2023/03/14 21:23:19
Sample name	GRH-YP202312717-XY-69-2	Calculation date and time	2023/03/23 10:22:57
Database name	2023-03-14.chd	Acquisition time [min]	0.000 - 20.000
Data name	RSLT4536	Sampling interval [msec]	100
Method name	PS-20230320	Cup number	10
Channel	RI	Calculation type	Molecular Weight

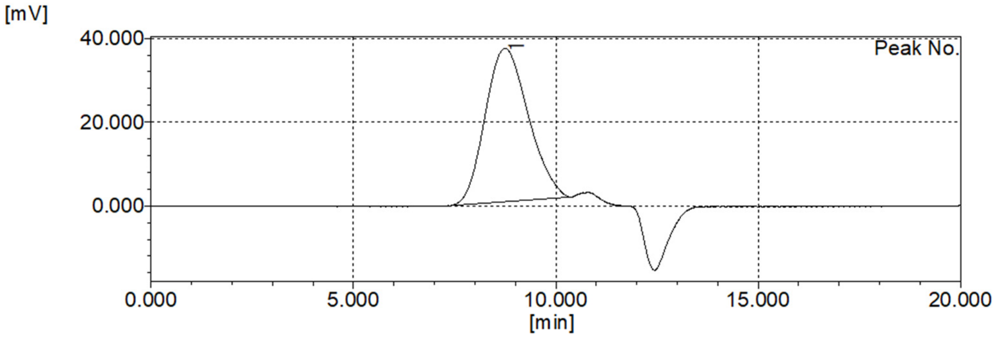

Result of molecular weight calculation (RI)

Peak 1 Base Peak

	[min]	[mV]	[mol]		
Peak start	7.417	0.090	80,809	Mn	6,634
Peak top	8.747	37.691	10,157	Mw	11,624
Peak end	10.332	2.093	857	Mz	18,235
				Mz+1	25,610
				Mv	11,624
Height [mV]			36.687	Mp	10,157
Area [mV*sec]			2713.487	Mz/Mw	1.569
Area% [%]			100.000	Mw/Mn	1.752
[eta]			11624.41022	Mz+1/Mw	2.203

Figure S24. SEC chart of the polymer obtained in Table 1, entry 2. (Mn = 6.6 kg·mol^{-1}, Mw/Mn = 1.75)

Header

Title		Data acquisition date and time	2023/03/14 21:43:20
Sample name	GRH-YP202312717-XY-70-1	Calculation date and time	2023/03/23 10:23:23
Database name	2023-03-14.chd	Acquisition time [min]	0.000 - 20.000
Data name	RSLT4537	Sampling interval [msec]	100
Method name	PS-20230320	Cup number	11
Channel	RI	Calculation type	Molecular Weight

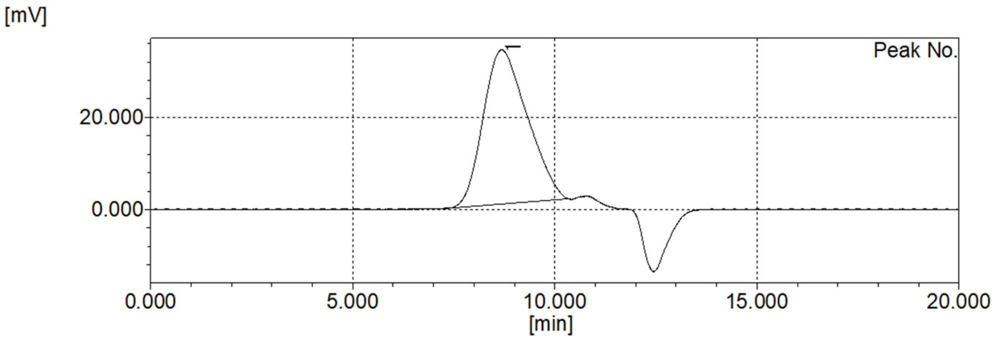

Result of molecular weight calculation (RI)

Peak 1 Base Peak

	[min]	[mV]	[mol]		
Peak start	7.440	0.359	77,922	Mn	6,536
Peak top	8.692	34.691	11,066	Mw	11,790
Peak end	10.313	2.374	882	Mz	18,593
				Mz+1	25,906
				Mv	11,790
Height [mV]			33.454	Mp	11,067
Area [mV*sec]			2532.876	Mz/Mw	1.577
Area% [%]			100.000	Mw/Mn	1.804
[eta]			11790.45198	Mz+1/Mw	2.197

Figure S25. SEC chart of the polymer obtained in Table 1, entry 3. (Mn = 6.5 kg·mol^{-1}, Mw/Mn = 1.80)

Header

Title		Data acquisition date and time	2023/03/14 22:03:21
Sample name	GRH-YP202312717-XY-71-1	Calculation date and time	2023/03/23 10:23:50
Database name	2023-03-14.chd	Acquisition time [min]	0.000 - 20.000
Data name	RSLT4538	Sampling interval [msec]	100
Method name	PS-20230320	Cup number	12
Channel	RI	Calculation type	Molecular Weight

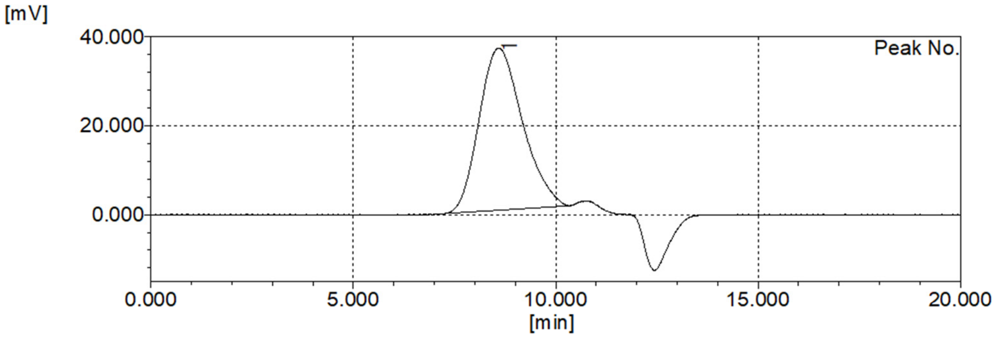

Result of molecular weight calculation (RI)

Peak 1 Base Peak

	[min]	[mV]	[mol]		
Peak start	7.232	0.229	107,832	Mn	7,973
Peak top	8.577	37.513	13,240	Mw	14,514
Peak end	10.383	2.020	791	Mz	22,955
				Mz+1	32,268
				Mv	14,514
Height [mV]			36.520	Mp	13,240
Area [mV*sec]			2721.306	Mz/Mw	1.582
Area% [%]			100.000	Mw/Mn	1.820
[eta]			14514.40895	Mz+1/Mw	2.223

Figure S26. SEC chart of the polymer obtained in Table 1, entry 4. (Mn = 8.0 kg·mol^{-1}, Mw/Mn = 1.82)

Header

Title		Data acquisition date and time	2023/03/14 22:23:23
Sample name	GRH-YP202312717-XY-71-2	Calculation date and time	2023/03/23 10:24:17
Database name	2023-03-14.chd	Acquisition time [min]	0.000 - 20.000
Data name	RSLT4539	Sampling interval [msec]	100
Method name	PS-20230320	Cup number	13
Channel	RI	Calculation type	Molecular Weight

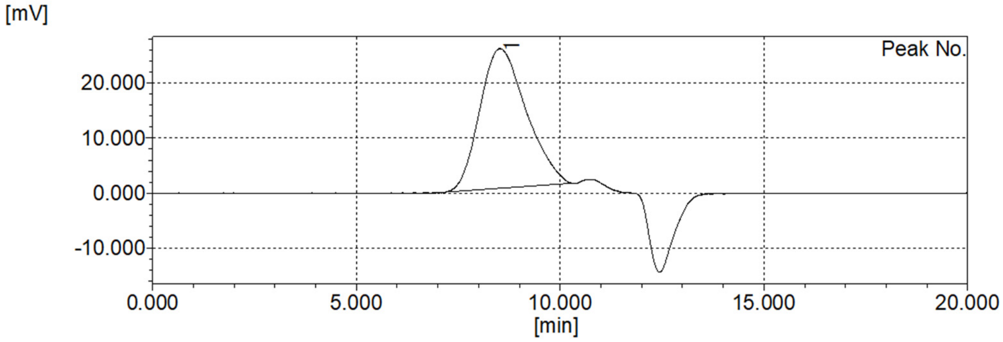

Result of molecular weight calculation (RI)

Peak 1 Base Peak

	[min]	[mV]	[mol]		
				Mn	8,251
Peak start	7.117	0.178	129,011	Mw	15,914
Peak top	8.523	26.312	14,388	Mz	26,097
Peak end	10.302	1.824	898	Mz+1	37,290
				Mv	15,914
Height [mV]			25.407	Mp	14,388
Area [mV*sec]			2008.475	Mz/Mw	1.640
Area% [%]			100.000	Mw/Mn	1.929
[eta]			15913.59598	Mz+1/Mw	2.343

Figure S27. SEC chart of the polymer obtained in Table 1, entry 5. (Mn = 8.3 kg·mol^{-1}, Mw/Mn = 1.93)

Header

Title		Data acquisition date and time	2023/03/14 22:43:24
Sample name	GRH-YP202312717-XY-72-1	Calculation date and time	2023/03/23 10:24:43
Database name	2023-03-14.chd	Acquisition time [min]	0.000 - 20.000
Data name	RSLT4540	Sampling interval [msec]	100
Method name	PS-20230320	Cup number	14
Channel	RI	Calculation type	Molecular Weight

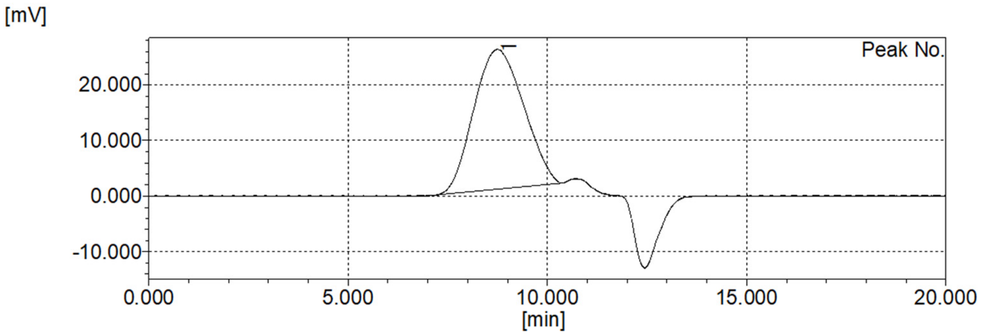

Result of molecular weight calculation (RI)

Peak 1 Base Peak

	[min]	[mV]	[mol]		
				Mn	6,496
Peak start	7.205	0.242	112,410	Mw	13,040
Peak top	8.738	26.441	10,289	Mz	23,004
Peak end	10.358	2.362	822	Mz+1	34,186
				Mv	13,040
Height [mV]			25.168	Mp	10,290
Area [mV*sec]			2179.761	Mz/Mw	1.764
Area% [%]			100.000	Mw/Mn	2.007
[eta]			13040.17051	Mz+1/Mw	2.622

Figure S28. SEC chart of the polymer obtained in Table 1, entry 6. (Mn = 6.5 kg·mol^{-1}, Mw/Mn = 2.00)

Header

Title		Data acquisition date and time	2023/03/22 23:25:00
Sample name	ZXH-YP202315344-xy-79-1	Calculation date and time	2023/03/23 10:04:27
Database name	2023-03-22.chd	Acquisition time [min]	0.000 - 20.000
Data name	RSLT4603	Sampling interval [msec]	100
Method name	PS-20230320	Cup number	15
Channel	RI	Calculation type	Molecular Weight

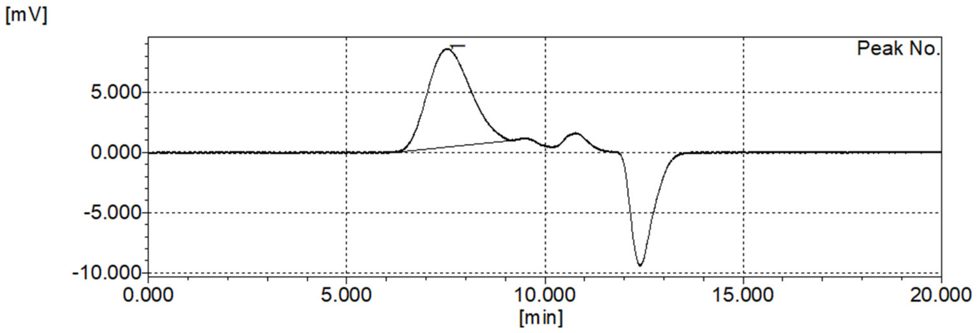

Result of molecular weight calculation (RI)

Peak 1 Base Peak

	[min]	[mV]	[mol]
Peak start	6.172	-0.006	563,115
Peak top	7.522	8.668	68,605
Peak end	9.165	0.985	5,290
Height [mV]			8.227
Area [mV*sec]			619.195
Area% [%]			100.000
[eta]			76566.14692

Mn	42,831
Mw	76,566
Mz	121,269
Mz+1	171,765
Mv	76,566
Mp	68,605
Mz/Mw	1.584
Mw/Mn	1.788
Mz+1/Mw	2.243

Figure S29. SEC chart of the polymer obtained in Table 1, entry 7. (Mn = 42.8 kg·mol^{-1}, Mw/Mn = 1.79)

Header

Title		Data acquisition date and time	2023/03/23 00:45:06
Sample name	ZXH-YP202315344-xy-74-2	Calculation date and time	2023/03/23 10:09:53
Database name	2023-03-22.chd	Acquisition time [min]	0.000 - 20.000
Data name	RSLT4607	Sampling interval [msec]	100
Method name	PS-20230320	Cup number	19
Channel	RI	Calculation type	Molecular Weight

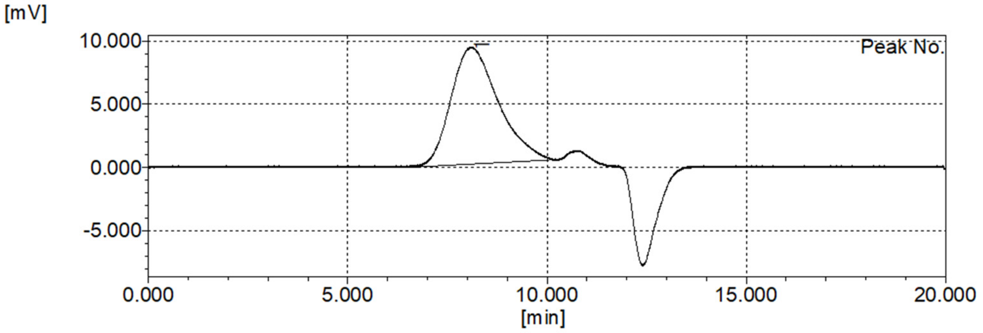

Result of molecular weight calculation (RI)

Peak 1 Base Peak

	[min]	[mV]	[mol]		
Peak start	6.597	0.024	290,255	Mn	13,907
Peak top	8.097	9.561	27,987	Mw	30,923
Peak end	10.225	0.623	1,013	Mz	54,687
				Mz+1	85,164
				Mv	30,923
Height [mV]			9.289	Mp	27,987
Area [mV*sec]			764.519	Mz/Mw	1.769
Area% [%]			100.000	Mw/Mn	2.224
[eta]			30922.87134	Mz+1/Mw	2.754

Figure S30. SEC chart of the polymer obtained in Table 1, entry 8. (Mn = 13.9 kg·mol^{-1}, Mw/Mn = 2.22).

Header

Title		Data acquisition date and time	2023/03/23 01:25:08
Sample name	ZXH-YP202315344-xy-75-1	Calculation date and time	2023/03/23 10:12:21
Database name	2023-03-22.chd	Acquisition time [min]	0.000 - 20.000
Data name	RSLT4609	Sampling interval [msec]	100
Method name	PS-20230320	Cup number	21
Channel	RI	Calculation type	Molecular Weight

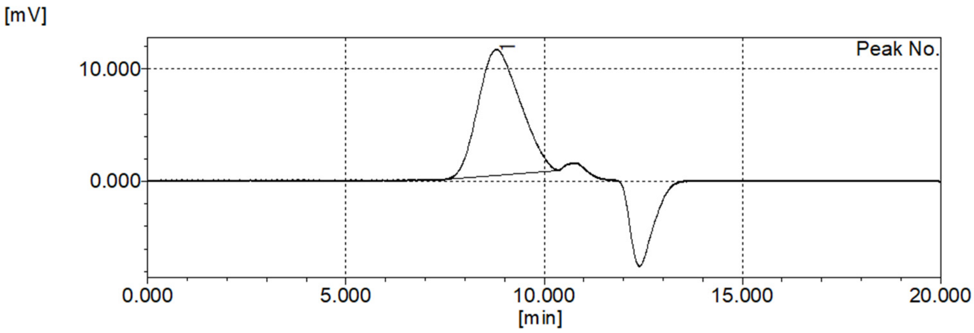

Result of molecular weight calculation (RI)

Peak 1 Base Peak

	[min]	[mV]	[mol]		
				Mn	5,883
Peak start	7.353	0.140	89,197	Mw	10,239
Peak top	8.797	11.790	9,395	Mz	16,155
Peak end	10.320	0.958	873	Mz+1	23,323
				Mv	10,239
Height [mV]			11.252	Mp	9,395
Area [mV*sec]			828.429	Mz/Mw	1.578
Area% [%]			100.000	Mw/Mn	1.740
[eta]			10239.01058	Mz+1/Mw	2.278

Figure S31. SEC chart of the polymer obtained in Table 1, entry 9. (Mn = 5.9 kg·mol^{-1}, Mw/Mn = 1.74)

Header

Title		Data acquisition date and time	2023/03/23 00:05:03
Sample name	ZXH-YP202315344-xy-75-2	Calculation date and time	2023/03/23 10:07:41
Database name	2023-03-22.chd	Acquisition time [min]	0.000 - 20.000
Data name	RSLT4605	Sampling interval [msec]	100
Method name	PS-20230320	Cup number	17
Channel	RI	Calculation type	Molecular Weight

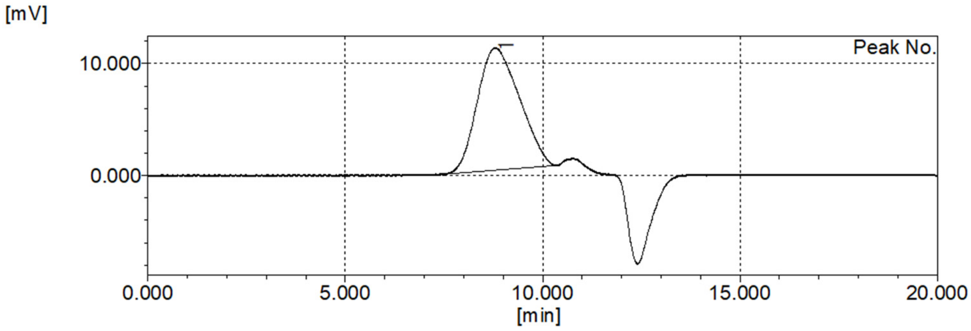

Result of molecular weight calculation (RI)

Peak 1 Base Peak

	[min]	[mV]	[mol]		
Peak start	7.390	0.097	84,240	Mn	5,820
Peak top	8.802	11.468	9,322	Mw	9,997
Peak end	10.358	0.913	822	Mz	15,650
				Mz+1	22,408
				Mv	9,997
Height [mV]			10.983	Mp	9,322
Area [mV*sec]			810.008	Mz/Mw	1.566
Area% [%]			100.000	Mw/Mn	1.718
[eta]			9996.70852	Mz+1/Mw	2.242

Figure S32. SEC chart of the polymer obtained in Table 1, entry 10. (Mn = 5.8 kg·mol^{-1}, Mw/Mn = 1.72)

Header

Title		Data acquisition date and time	2023/03/22 23:45:02
Sample name	ZXH-YP202315344-xy-80-2	Calculation date and time	2023/03/23 10:06:48
Database name	2023-03-22.chd	Acquisition time [min]	0.000 - 20.000
Data name	RSLT4604	Sampling interval [msec]	100
Method name	PS-20230320	Cup number	16
Channel	RI	Calculation type	Molecular Weight

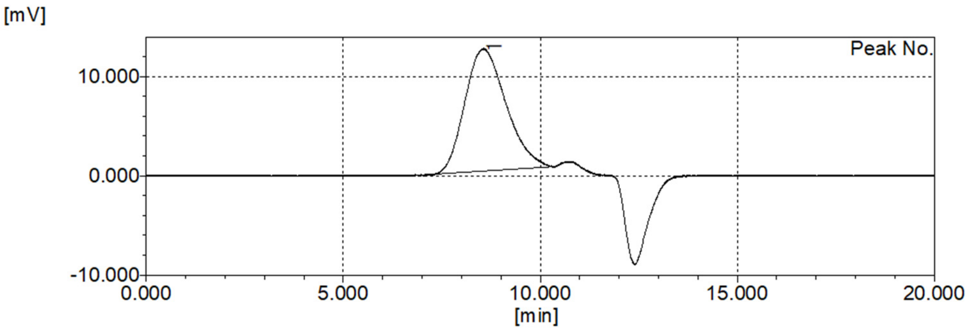

Result of molecular weight calculation (RI)

Peak 1 Base Peak

	[min]	[mV]	[mol]		
Peak start	7.100	0.109	132,408	Mn	8,536
Peak top	8.545	12.825	13,910	Mw	15,213
Peak end	10.252	0.891	971	Mz	23,837
				Mz+1	34,288
				Mv	15,213
Height [mV]			12.357	Mp	13,910
Area [mV*sec]			896.117	Mz/Mw	1.567
Area% [%]			100.000	Mw/Mn	1.782
[eta]			15212.56104	Mz+1/Mw	2.254

Figure S33. SEC chart of the polymer obtained in Table 1, entry 11. (Mn = 8.5 kg·mol^{-1}, Mw/Mn = 1.78)

Header

Title		Data acquisition date and time	2023/03/23 01:05:06
Sample name	ZXH-YP202315344-xy-76-1	Calculation date and time	2023/03/23 10:11:17
Database name	2023-03-22.chd	Acquisition time [min]	0.000 - 20.000
Data name	RSLT4608	Sampling interval [msec]	100
Method name	PS-20230320	Cup number	20
Channel	RI	Calculation type	Molecular Weight

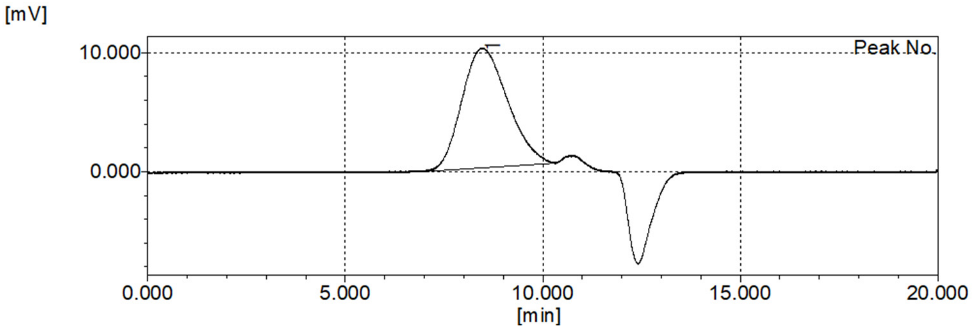

Result of molecular weight calculation (RI)

Peak 1 Base Peak

	[min]	[mV]	[mol]
Peak start	6.872	-0.015	189,036
Peak top	8.457	10.439	15,964
Peak end	10.375	0.734	801
Height [mV]			10.115
Area [mV*sec]			812.125
Area% [%]			100.000
[eta]			18144.98930

Mn	9,135
Mw	18,145
Mz	31,371
Mz+1	49,274
Mv	18,145
Mp	15,965
Mz/Mw	1.729
Mw/Mn	1.986
Mz+1/Mw	2.716

Figure S34. SEC chart of the polymer obtained in Table 1, entry 12. (Mn = 9.1 kg·mol^{-1}, Mw/Mn = 1.99)

Header

Title		Data acquisition date and time	2023/04/19 16:53:10
Sample name	XY-YP202322263-85-1	Calculation date and time	2023/04/19 19:18:02
Database name	2023-04-19.chd	Acquisition time [min]	0.000 - 20.000
Data name	RSLT4790	Sampling interval [msec]	100
Method name	PS-20230320	Cup number	6
Channel	RI	Calculation type	Molecular Weight

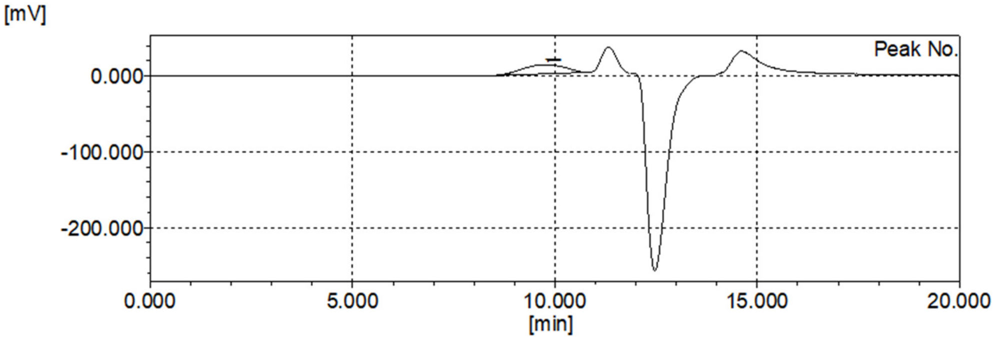

Result of molecular weight calculation (RI)

Peak 1 Base Peak

	[min]	[mV]	[mol]
Peak start	8.382	0.279	17,945
Peak top	9.700	14.990	2,296
Peak end	10.825	4.875	397
Height [mV]			12.231
Area [mV*sec]			889.844
Area% [%]			100.000
[eta]			2903.60267

Mn	1,830
Mw	2,904
Mz	4,475
Mz+1	6,271
Mv	2,904
Mp	2,297
Mz/Mw	1.541
Mw/Mn	1.587
Mz+1/Mw	2.160

Figure S35. SEC chart of the polymer obtained in Table 1, entry 10 after treatment of tri-*n*-butylphosphine. (Mn = 1.8 kg·mol^{-1}, Mw/Mn = 1.59)
